# Cholinergic axons regulate type I acini in salivary glands of *Ixodes ricinus* and *Ixodes scapularis* ticks

**DOI:** 10.1038/s41598-020-73077-1

**Published:** 2020-09-29

**Authors:** Lourdes Mateos-Hernandéz, Baptiste Defaye, Marie Vancová, Ondrej Hajdusek, Radek Sima, Yoonseong Park, Houssam Attoui, Ladislav Šimo

**Affiliations:** 1 UMR BIPAR, INRAE, Ecole Nationale Vétérinaire d’Alfort, ANSES, Université Paris-Est, Maisons-Alfort, France; 2 Faculté de Pharmacie, Université de Limoges, Limoges, France; 3 Biology Centre, Institute of Parasitology, Czech Academy of Sciences, České Budejovice, Czech Republic; 4 Faculty of Science, University of South Bohemia, České Budejovice, Czech Republic; 5 Department of Entomology, Kansas State University, 123 Waters Hall, Manhattan, KS USA; 6 UMR Virologie, INRAE, Ecole Nationale Vétérinaire d’Alfort, ANSES, Université Paris-Est, Maisons-Alfort, France; 7 Present Address: UMR SPE 6134 CNRS, Université de Corte Pascal Paoli, Corse, France

**Keywords:** Biochemistry, Molecular biology, Neuroscience, Physiology

## Abstract

Regulatory factors controlling tick salivary glands (SGs) are direct upstream neural signaling pathways arising from the tick’s central nervous system. Here we investigated the cholinergic signaling pathway in the SG of two hard tick species. We reconstructed the organization of the cholinergic gene locus, and then used in situ hybridization to localize mRNA encoding choline acetyltransferase (ChAT) and vesicular acetylcholine transporter (VAChT) in specific neural cells in the *Ixodes* synganglion. Immunohistochemical staining revealed that cholinergic axonal projections exclusively reached type I acini in the SG of both *Ixodes* species. In type I acini, the rich network of cholinergic axons terminate within the basolateral infoldings of the lamellate cells. We also characterized two types (A and B) of muscarinic acetylcholine receptors (mAChRs), which were expressed in *Ixodes* SG. We pharmacologically assessed mAChR-A to monitor intracellular calcium mobilization upon receptor activation. In vivo injection of vesamicol—a VAChT blocker—at the cholinergic synapse, suppressed forced water uptake by desiccated ticks, while injection of atropine, an mAChR-A antagonist, did not show any effect on water volume uptake. This study has uncovered a novel neurotransmitter signaling pathway in *Ixodes* SG, and suggests its role in water uptake by type I acini in desiccated ticks.

## Introduction

The European castor-bean tick *Ixodes ricinus*, and the North American black-legged tick *Ixodes scapularis*, are the principal vectors of spirochetes causing Lyme disease^1,2^. These allopatric hard tick species have a life cycle spanning three years. *Ixodes*, ticks take a blood meal during each of their hematophagous stages, (larva, nymph, and female), to facilitate molting into the next stage or lay eggs^3^. A tick’s parasitic stage—characterized by active blood uptake from a host—is often recognized as the biological hallmark, however they spend the majority of their life cycle in a non-parasitic fasting state. During both periods, their survival depends on effectively maintaining water homeostasis.

Tick salivary glands (SGs) play crucial osmoregulatory roles during both the on- and off-host stages and their activity directly reflects the biological success of ticks in the environment^4–7^. In female hard ticks, grape-like SG clusters are composed of three types of spherical acini (type I, II, and III hereafter). Agranular type I acini are almost exclusively located at the anterior part of the main salivary duct, while the granular type II and III acini are associated with more distally-located secondary and tertiary ducts, respectively^8–10^. Studies examining tick SG physiology have primarily focused on the on-host tick feeding period, during which the secretion activities of this tissue are the most apparent. In particular, catecholamines dopamine and norepinephrine, and cholinomimetic agent pilocarpine, have been shown to be powerful activators of tick SG secretions^11–15^. Subsequently, recent technological developments in the post-genomic era have led to several breakthroughs in understanding the molecular physiology of tick SG, providing solid evidence that saliva production is under the control of autocrine/paracrine dopamine and synaptic release of neuropeptides in both granular type II and III acini^7,11,16–19^. Furthermore, it has been clearly documented that these structures are highly innervated by different axons arising from the synganglion, the tick’s central nervous system^17,19–24^. Generally, there have been fewer investigations into type I acini, that unique morphology and proposed role make them far different from the functionally similar types II and III^7,8,10^. Over the last four decades, multiple reports have primarily associated type I acini activity with the fasting period, when ticks often undergo stress due to desiccation^25–28^. In dry conditions, tick secrets Na^+^/K^+^-rich hygroscopic saliva onto their mouthpart surfaces, forming a crystalized matrix. When environmental humidity increases, this salty deposit is deliquesced with water vapor and subsequently ingested. Then, ingested water is absorbed by type I acini, thus keeping the tick hydrated^26,27,29–31^. Moreover, type I acini also appear to have an additional role in sodium ion uptake from the primary saliva generated by types II and III acini during tick feeding^7,25,32^.

Mediation of tick SG fluid secretion by cholinomimetic drug pilocarpine, has been considered as a common saliva induction process in many tick species worldwide^14,33,34^. Injection of the muscarinic acetylcholine receptor (mAChR) agonist, pilocarpine, into partially-fed hard tick females induces robust long-lasting SG secretion, whereas it fails to induce saliva from isolated SG. Subsequently, it has been suggested that the synganglion is essential for pilocarpine-mediated SG fluid secretion^14,33^. To date, only indirect evidence indicate the presence of mAChR in ticks^33,35^. In addition, the molecular characterization of choline acetyltransferase (ChAT), the enzyme involved in acetylcholine (ACh) synthesis, and the vesicular acetylcholine transporter (VAChT), responsible for loading ACh into secretory granules in pre-synaptic cells^36,37^ remain understudied.

In the present study, we investigated the organization of cholinergic gene locus in two *Ixodid* tick species. Distribution of cholinergic neurons along their axonal projections reaching acini type I in SG was also examined. Two different types of mAChRs were characterized and functionally tested. Subsequently, an in vivo experiment was conducted in order to determine, whether the cholinergic axons regulate the type I SG acini activities in desiccated ticks.

## Materials and methods

### Experimental animals and chemicals

We confirm that all experiments were performed in accordance with relevant guidelines and regulations. *Ixodes ricinus* ticks were obtained from the colonies of UMR-BIPAR, Maisons-Alfort, France or the Institute of Parasitology, Biological Centre of Czech Academy of Sciences, Czech Republic. Unfed adult *I. scapularis* ticks were sourced from a tick-rearing facility at Oklahoma State University. Approximately 30 ticks, males or females, were each kept individually in a polypropylene tube containing a piece of filter paper (3 × 1 cm). Vials were maintained with a light–dark (12 h/12 h) cycle in a glass desiccator with > 97% relative humidity at 22 °C. Ticks were fed on New Zealand rabbits^38^ and the protocol was approved by the ComEth Anses/ENVA/UPEC Ethics Committee for Animal Experimentation, (permit No. E 94 046 08). The protocol to feed ticks on Guinea pigs was approved by the Committee on the Ethics of Animal Experimentation of the Institute of Parasitology and of the Departmental Expert Committee for the Approval of Projects of Experiments on Animals of the Czech Academy of Sciences (permit No. 165/2010).

Chemicals used in this study included: acetylcholine chloride (Sigma), (+)-muscarine chloride (Sigma), pilocarpine hydrochloride, dopamine hydrochloride, (±)-octopamine hydrochloride (Sigma), (±)-epinephrine hydrochloride (Sigma), atropine (Sigma), and (±)-vesamicol hydrochloride (Tocris).

### Molecular cloning and sequence analyses

BLAST searches of the *I. scapularis* genome^39^ were performed within Vectorbase and NCBI databases (www.vectorbase.org; www.ncbi.nlm.nih.gov). Full-length open reading frames (ORFs) of putative *machr-a* and -*b* were identified using *Drosophila* *melanogaster* mAChR-A and -B protein sequences, JQ922421.1 and JX028235.1 respectively. To obtain the full ORFs for both receptors, the primers (Supplementary Table S1) were designed based on the *Ixodes* genomic sequence just before putative translation initiation signal and after the stop codon.

The *D. melanogaster* ChAT and VAChT protein sequences NP_996239.2; NP_477138.1, respectively, were used to query the *Ixodes* databases. This search retrieved partial sequences of the putative *I. scapularis chat* and *vacht* genes. *I. ricinus* transcript sequence of *chat* (in-house *I. ricinus* transcript project, Czech Republic, Biology Centre, České Budejovice) was used to complement the full-length exon–intron structure of the *chat I. scapularis* gene. Shared exon 1 of *chat* and *vacht* and mutually exclusive exons 13 and 14 of *chat* were experimentally confirmed by reverse transcription polymerase chain reaction (RT-PCR) using cDNA of *I. scapularis* or *I. ricinus* synganglia. The short PCR products of the *chat/vacht* shared exon 1 was commercially sequenced (Eurofins). The PCR amplicon of *chat* exons 9–14, *vacht* exon 2 and both full length ORFs of *machr-a* and *b* were inserted into the pGEM-T Easy vector (Promega) followed by transformation of competent DHα bacteria (prepared using the Mix & Go kit, Zymo Research). Plasmid DNA was purified using the Nucleospin Plasmid kit (Macherey–Nagel). Recombinant plasmids were commercially sequenced (Eurofins). For the primers information see Supplementary Table S1.

The exon–intron structure of *chat* gene was manually analyzed using Vector NTI software (Invitrogen) and graphical images were generated using the Exon–Intron Graphic Marker version 4 (WormWeb.org). Putative translations were aligned using the Clustal program^40^. For phylogenetics, a neighbor-joining tree was constructed in MEGA7 software^41^, with 500 bootstrap replications. The prediction of transmembrane segments in VAChT, mAChR-A, and mAChR-B were performed using TOPCONS software (https://topcons.cbr.su.se/pred/)^42^. Graphical visualization of transmembrane receptors was performed in Protter 1.0 (https://wlab.ethz.ch/protter/start/).

### Whole-mount immunohistochemistry

We followed validated protocols previously described by Šimo et al.^19,23,43^, which were successfully used for tick-tissue immunohistochemistry (IHC). Briefly, *Ixodes* SGs and synganglia were dissected from unfed adults and fixed with Bouin’s solution for 2 h at room temperature (RT), then washed with PBS + 0.3% Triton X-100 (PBST). Tissues were incubated with monoclonal anti-mouse antibody against *D. melanogaster* ChAT (ChAT4B1, Developmental Studies Hybridoma Bank University of Iowa) diluted 1:500 in PBST for 3 days at 4 °C. After washes in PBST, the specimens were incubated overnight at 4 °C with a goat anti-mouse Alexa 488 conjugated secondary antibody (Life technologies) diluted at 1:1000. Samples were mounted in Prolong Antifade Diamond Mountant containing DAPI (Life Technologies) and analyzed by inverted confocal microscopy Zeiss LSM 700. Image adjustment was performed in Adobe Photoshop CS6 (Adobe System Incorporate, 2012). For neuronal cells we used nomenclatures as per Šimo et al.^23^. The first two letters refer to the position of each neuron in a specific lobe of the synganglion, prothocerebral (Pc), pedal 1–4 (Pd_1–4_), opisthosomal (Os), preoesophageal (Pe) or postoesophageal (Po), and the letters that follow refer to the anatomical location of the neuron: dorsal (D), ventral (V), anterior (A), posterior (P), medial (M) or lateral (L). Neurons innervating acini of salivary glands were labeled SG.

### In situ hybridization

We followed the validated in situ hybridization (ISH) protocol previously developed by Šimo et al.^19^ for tick synganglia. Briefly, single-stranded digoxygenin-labelled DNA probes for *chat* and *vacht* (954 and 505 bp long, respectively), were prepared using asymmetric PCR using the DIG probe synthesis kit (Roche Diagnostic, Germany). To generate antisense probes, only the reverse primer was used. As for the control sense probes (see Supplementary Fig. S1), we only used the forward primer. For the primers information see Supplementary Table S1. Synthetized probes were gel-purified and stored at − 20 °C. Synganglia of unfed *I. ricinus* females were dissected in cold PBS and fixed with 4% paraformaldehyde for 3 h at RT. Cell membrane permeability was enhanced by treating tick synganglia with Proteinase K (New England BioLabs). Specimens were incubated with single-stranded DNA probes for 27 h at 48 °C, followed by incubation with mouse anti-digoxygenin/AP (Alkaline phosphatase; Roche Diagnostics, Germany) overnight at 4 °C. Hybridized probes were detected following the addition of substrate/chromogen ready-to-use NBT-BCIP tablets (Roche Diagnostics, Germany). Finally, samples were incubated 5 min in 50% glycerol and subsequently mounted into 100% glycerol and observed by light microscopy (Olympus BX53). Images were assembled in Adobe Photoshop CS6.

### Transmission electron microscopy

For immunogold labeling of SG we used methodology successfully used in our recent study by Vancová et al.^24^. Concisely, SGs intended for ChAT immunolocalization were dissected from unfed *I. ricinus* females and fixed in a mixture containing 4% formaldehyde and 0.1% glutaraldehyde in 0.1 M HEPES for 1 h at RT. After washing in HEPES buffer with 0.02 M glycine, specimens were cryoprotected in 2.3 M sucrose for 72 h at 4 °C and frozen by plunging into liquid nitrogen. Ultrathin cryosections were cut at − 100 °C, picked up with 1.15 M sucrose/1% methylcellulose solution (25 cP, Sigma). Sections were incubated for 30 min at RT in a blocking buffer (BB) composed of 1% fish skin gelatin (Sigma) and 0.05% Tween 20 and labelled for 1 h at RT with anti-mouse antibody against *D. melanogaster* ChAT diluted 1:30 in BB. After washing in BB, sections were incubated with goat anti-mouse IgG coupled with 5 nm gold nanoparticles (British Biocell International) diluted 1:40 in BB. After 1 h, sections were washed in HEPES, post fixed for 5 min in 2.5% glutaraldehyde, washed in dH_2_O, then contrasted/embedded using a mixture of 2% methylcellulose and 3% aqueous uranyl acetate solution (9:1).

For ultrastructural studies we slightly modified the protocol previously used by Bílý et al.^44^. SGs isolated from a partially-fed ticks (5 days, guinea pig) were frozen under high pressure (Leica EM PACT2) in the presence of 20% bovine serum albumin. Freeze substitution was performed in 2% OsO_4_ in 100% acetone (− 90 °C, 96 h). Then the temperature was increased to − 20 °C (5 °C/h) and after 24 h up to 4 °C (5 °C/h). Samples were washed three times for 15 min in 100% acetone, infiltrated, and embedded in EMBed 812 resin (EMS). Ultrathin sections were stained in ethanolic uranyl acetate for 30 min and lead citrate for 20 min. All samples were observed using a JEOL 1010 transmission electron microscope.

### Functional receptor assays

The full-length ORF of mAChR-A was inserted into the expression plasmid pcDNA3/Zeo(+) (Invitrogen). mAChR-A was transiently expressed with the aequorin reporter (human cytoplasmic aequorin^45^) in Chinese hamster ovary (CHO-K1, Sigma) cells to monitor intracellular calcium mobilization-triggered bioluminescence upon activation of the receptor^18,43^. The assay was performed in opaque 96-well microplates (Nunc) using the Fluostar Omega microplate reader (BMG Labtech). Data obtained were analyzed in Excel (Microsoft Office) and the dose response curves, including the half maximum response values (EC_50_ or IC_50_), were calculated using the GraphPad Prism 5 software package (GraphPad Software, La Jolla California USA).

Cells were simultaneously co-transfected with pcDNA3/Zeo(+)/mAChR, pcDNA3/Zeo(+)/human cytoplasmic aequorin, and pcDNA3.1(+)/wild type human G protein alpha 15 subunit (G_α15(16)_, cDNA Resource Center, Bloomsburg University of Pennsylvania) constructs. The use of chimeric G_α15(16)_ subunit is advocated due to its high efficiency when linking calcium mobilization signaling pathways to transfected G_αi/o_ coupled receptors^46^. The receptor’s activity was also assessed in the absence of the G_α15(16)_ subunit. Cells were pre-equilibrated with coelenterazine h (Promega) for 3 h at RT. Various doses of agonist ligands in 50 μL were added into each well followed by injection of a 50 μl cell suspension (containing approximately 15,000 cells). Immediately after the injections, changes in luminescence were monitored for 20 s and their integrated values over time were normalized to the largest positive control response in each plate (10 μM ACh) after background subtractions. For the antagonist assay, cells were pre-incubated with different doses of atropine (an mAChR-A agonist) in a 96-well microplate at RT for 5 min and subsequently treated with 10 μM ACh. Emission of luminescence (over 20 s) was measured immediately after injecting ACh and time integrated values were normalized to the lowest response (highest dose of atropine) in each plate after subtracting background.

Mock transfections using only the reporter and G_α15(16)_ were used as negative controls. At least three biological replicates were performed for each assay, with two wells per sample for each given ligand dose. Conditions for handling cell lines and transfection details are provided in Šimo et al.^18^. Information regarding the mAChR-B functional assay, which monitors cAMP elevations, are provided in the Supplementary Methods.

### Tissue-specific and quantitative real-time reverse transcriptase PCR (qRT-PCR)

Total RNA for tissue-specific PCR was extracted from different tick tissues such as: SGs, synganglia, Malpighian tubules, ovaries, tracheas, and intestines of partially fed (6 days) *I. ricinus* females. In addition, only the dorsal part of the cuticle and carcass (ventral cuticle with legs, muscles, and fat bodies) was also used for RNA extraction. Total RNA was extracted using Trizol reagent (Invitrogen). Reverse transcription was performed using Superscript III according to the manufacturer’s protocol (Invitrogen) in presence of oligo(dT) primers, and was followed by classical PCR amplification. For qRT-PCR, the synganglia and SGs of unfed *I. ricinus* females maintained in either 98% or 25% relative humidity (RH) for 30 h were used. The dissected tissues from 10 (first replication) and 20 (second replication) individuals were pooled for RNA extraction using the RNA micro kit (Qiagen). Real-time PCR was performed in a LightCycler 480 II (Roche) using SYBR premix Ex Taq (Roche). The ribosomal protein S4 (GenBank Accession number DQ066214) was used as a reference gene^47^. mRNA level was quantified using the ΔΔCt method, corrected by the amplification efficiency of each target gene, and expressed as a fold difference^48^. Data were analyzed by Microsoft Excel and final graphs were prepared in GraphPad Prism 5 (GraphPad Software, La Jolla California, USA). Statistics for the qRT-PCR values were calculated using a two-tailed *t*-test for minimum of three technical and two experimental replicates.

### Tick fluid ingestion assay

We slightly modified the methods previously used by Kim et al.^25^. Prior to the experiments, ticks were exposed to severe dehydrating conditions of 28 °C and 25% RH for 30 h. To investigate the physiological function of cholinergic axons reaching the SG type I acini, we injected dehydrated ticks with atropine, the mAChR-A antagonist or/and vesamicol, the VAChT inhibitor that reduces ACh uptake into secretory vesicles in presynaptic cholinergic axon terminals. Then, 50 nl of 100 μM drug(s) in PBS or PBS itself were injected into the ventral idiosoma of dehydrated *Ixodes* females using a nano-injector (Drummond) connected to a micro-syringe pump controller (Micro 4, WPI). After injection, ticks were maintained under dehydrating conditions for an additional 30 min. They were then placed upside down on double-sided sticky tape and their hypostomes were connected to a glass microcapillary tube (volume 1 μl, length 32 mm, Sigma) filled with water. Ticks were allowed to drink for one hour and the final fluid volume in the microcapillary tube was measured using a grid under a microscope. For statistical analyses we used a two-tailed *t*-test in GraphPad Prism 5 to determine significant differences between control (PBS-injected ticks) and treated groups. Two biological replications were performed.

## Results

### Organization of the cholinergic gene locus: ChAT and VAChT

**Figure 1:**
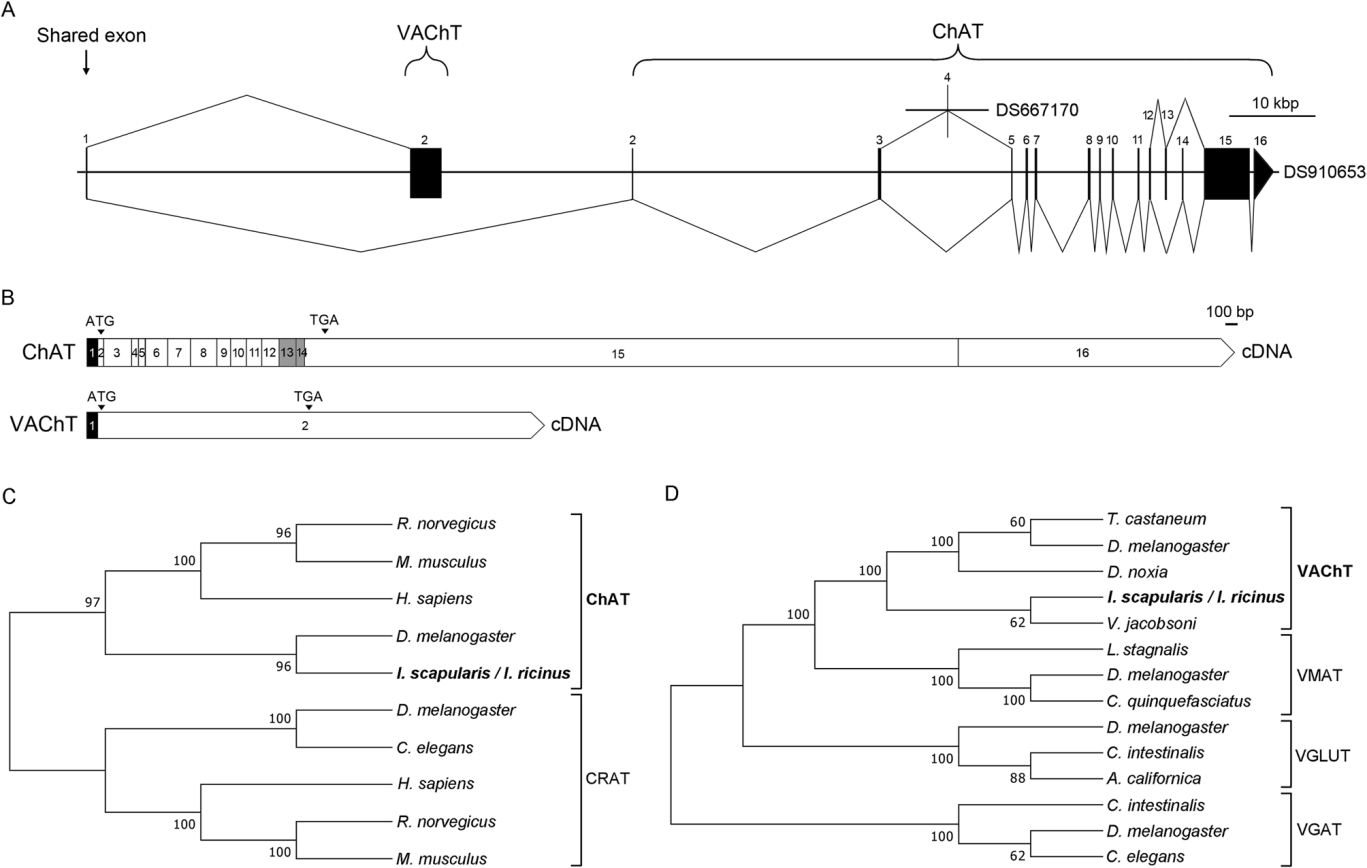
(**A**) Structure of the *I. scapularis* cholinergic gene locus (scaffold DS910653 is to scale). Horizontal lines represent introns, while vertical lines and black boxes represent exons (numbered). Splicing between exons is indicated with diagonal lines. Exon 1 is shared by both ChAT and VAChT. The second exon of VAChT (that spans its entire ORF) lies within the first intron of ChAT. Exon 4 is located in scaffold DS667170 (for more information see Supplementary Fig. S4). Exons numbered 13 and 14 are the two mutually exclusive exons. Graphical image was generated using the Exon–Intron Graphic Marker version 4 (https://wormweb.org/exonintron). (**B**) cDNA schematics of ChAT and VAChT indicating the proportional lengths of each exon (numbers). The black color indicates the region encoded by the shared exon. The grey color in ChAT (exons 13 and 14) indicate the two possible ChAT transcript variants (A and B) based on the mutually exclusive exons (one or the other, but not both) shown in (**A**). ATG and TAG indicate the positions of the initiation and stop codon respectively. (**C**,**D**) Phylogenetic relationships of ChAT (**C**) and VAChT (**D**) of *I. ricinus/I. scapularis*. *CRAT* carnitine acetyltransferase, *VMAT* vesicular monoamine transporter, *VGLUT* vesicular glutamate transporter, *VGAT* vesicular GABA transporter. GenBank Accession numbers are: *Rattus norvegicus* ChAT*,* XM_017600025.1; *Mus musculus* ChAT, NM_009891.2; *Homo sapiens* ChAT, AF305907.1; *D. melanogaster* ChAT, NM_057656.3; *I. scapularis* ChAT isoform A, MT669643 or *I. ricinus* ChAT isoform A, MT669641; *D. melanogaster* CRAT, NM_001300266.1; *Caenorhabditis elegans* CRAT, NM_064320.4; *H. sapiens* CRAT, NM_000755.5; *R. norvegicus* CRAT, NM_001004085.2; *M. musculus* CRAT, NM_007760.3; *Tribolium castaneum* VAChT, XM_970406.3; *D. melanogaster* VAChT, NP_477138.1; *Diuraphis noxia*, XP_015365490.1; *I. scapularis* VAChT MT669645 or *I. ricinus* VAChT, MT669644; *Varroa jacobsoni* VAChT, XM_022846413.1; *Lymnaea stagnalis* VMAT, AF484094.1; *D. melanogaster* VMAT, NM_001274027.2; *Culex quinquefasciatus* VMAT, XM_001849217.1; *D. melanogaster* VGLUT, NM_001273010.1; *Ciona intestinalis* VGLUT, NM_001128885.1; *Aplysia californica* VGLUT, NM_001310491.1; *C. intestinalis* VGAT, NM_001032573.1; *D. melanogaster* VGAT, NM_137094.3; and *C. elegans* VGAT, NM_066854.6. The phylogenetic threes in C and D were constructed using neighbor-joining method in MEGA7 software, with 500 bootstrap replications.

Homology searches were performed using BLAST algorithms from the NCBI (https://www.ncbi.nlm.nih.gov) and Vectorbase (www.vectorbase.org) databases. The search for ChAT revealed uncorrected transcripts corresponding to a putative *Ixodes* ChAT: XM_029980779.1 (*I. scapularis* putative mRNA predicted from genome sequence), ISCW022171-RA (*I. scapularis* transcript, gene set IscaW1.6), ISCI022171-RA (ISE6 cells transcript database, gene set IscaI1.0), and GBBN01014222.1 (assembled transcriptome database of *I. scapularis* female synganglia). All sequences were missing 5′ and 3′ prime ends. Furthermore, multiple discrepancies were found in their protein alignments (see Supplementary Fig. S2). The BLAST search identified a putative *Ixodes* VAChT transcript, ISCW022169-RA. A combination of computational and experimental annotation was used to identify the full-length sequence of the *I. scapularis* cholinergic gene locus (Fig. 1A). BLAST searches of both the putative ChAT and VAChT transcripts against the *I. scapularis* genome sequence confirmed their relationship to the DS910653 scaffold, except for a short ChAT exon 4 which aligned to the DS667170 scaffold (negative reading frame, Fig. 1A). To describe the exon–intron structure of the *I. scapularis* cholinergic gene locus, we employed manual annotation using *I. ricinus* ChAT and VAChT obtained from transcriptomic data (*I. ricinus* transcript project, in-house database). In the *I. scapularis* genome sequence, we identified a total of 16 and 2 exons (including a shared exon) for ChAT and VAChT, respectively (Fig. 1A,B). A shared exon (Fig. 1A) of ChAT and VAChT with a total length of 82 bp was experimentally confirmed by RT-PCR. The second exon of VAChT, encoding the ORF, lies within the first ChAT intron (Fig. 1A). Using RT-PCR, we confirmed that exons 13 and 14 of ChAT are mutually-exclusive spliced exons (one or the other, Fig. 1A, also see Supplementary Fig. S3). The ChAT sequences (Supplementary Fig. S4) were deposited into the GenBank database for *I. scapularis* transcript variant A and B (Accession numbers MT669643 and MT669646, respectively) and for *I. ricinus* transcript variant A and B (Accession numbers MT669641 and MT669642, respectively). VAChT transcripts for both *I. scapularis* and *I. ricinus* were also deposited into GenBank (Accession numbers MT669645 and MT669644, respectively).

Phylogenetic analyses of ChAT and VAChT protein sequences showed a clear evolutionary relationship with other arthropods and/or mammalian orthologues (Fig. 1C,D). The protein sequence of VAChT was predicted to contain 12 putative transmembrane domains typical of the VAChT family (Supplementary Fig. S5). *I. scapularis* and *I. ricinus* protein sequences of ChAT share 97.8% (isoform A), 97.7% (isoform B) and VAChT 99.1% identity.

### ChAT and VAChT in tick synganglion

**Figure 2:**
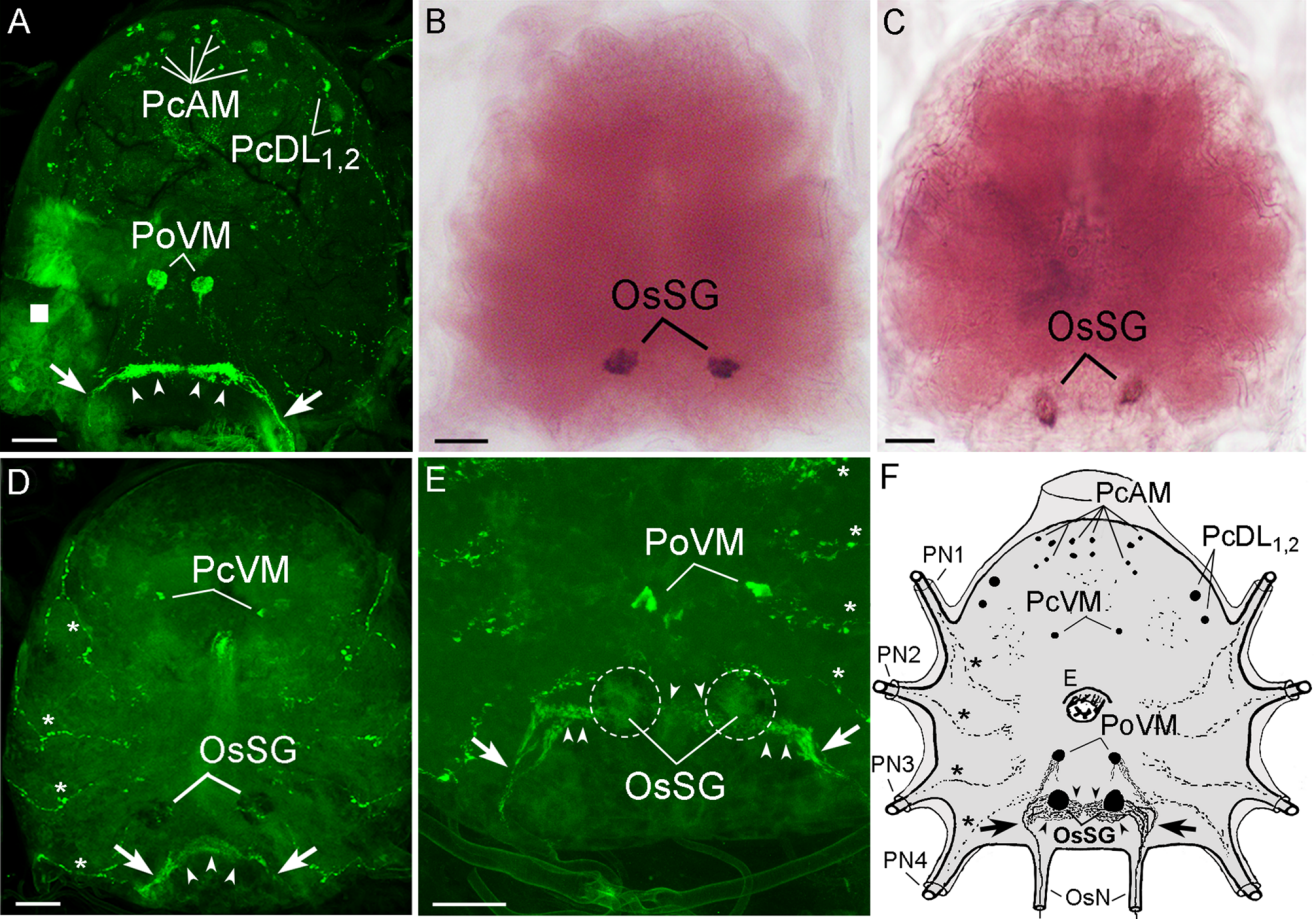
Whole-mounted immunohistochemistry (IHC) of ChAT (**A**,**D**,**E**) and whole-mounted in situ hybridization (ISH) of *chat* and *vacht* (**B**,**C**) in synganglia of unfed *Ixodes* females. (**A**) The synganglion of *I. scapularis* stained with antibody against *D. melanogaster* ChAT. (**B**,**C**) ISH of *chat* (**B**) and *vacht* (**C**) using their representative anti-sense probes in the synganglion of *I. ricinus*. (**D**) Ventral side of the *I. ricinus* synganglion after ISH of *chat* followed by IHC on the same specimen. Note that the dark spots are the SGs innervating opistosomal neurons (OsSG) visualized in ISH as seen in the confocal image. (**E**) Detail of the ventral posterior part of the synganglion (ISH followed by IHC) highlighting the OsSG neurons (in dotted circles) and associated axons. For a more detailed view see Videos 1 and 2. (**F**) Schematic illustration of the *Ixodes* synganglion highlighting the observed cholinergic neurons and their axons. Arrowheads—axonal clusters likely belonging to the OsSG neurons; arrows—axons exiting the OsSG cells and entering the opistosomal nerves (OsN), asterisks—lateral pedal axons, PN1-4—pedal nerves 1–4, E—esophagus; white square in (**A**) shows the disrupted part of the synganglion that facilitated the diffusion of the antibody inside the tissue. For the nomenclature of detected neurons see the section Whole-mount immunohistochemistry in the Material and methods. Scale bars are 50 μm. For ISH negative controls see Supplementary Fig. S2.

**Figure 3:**
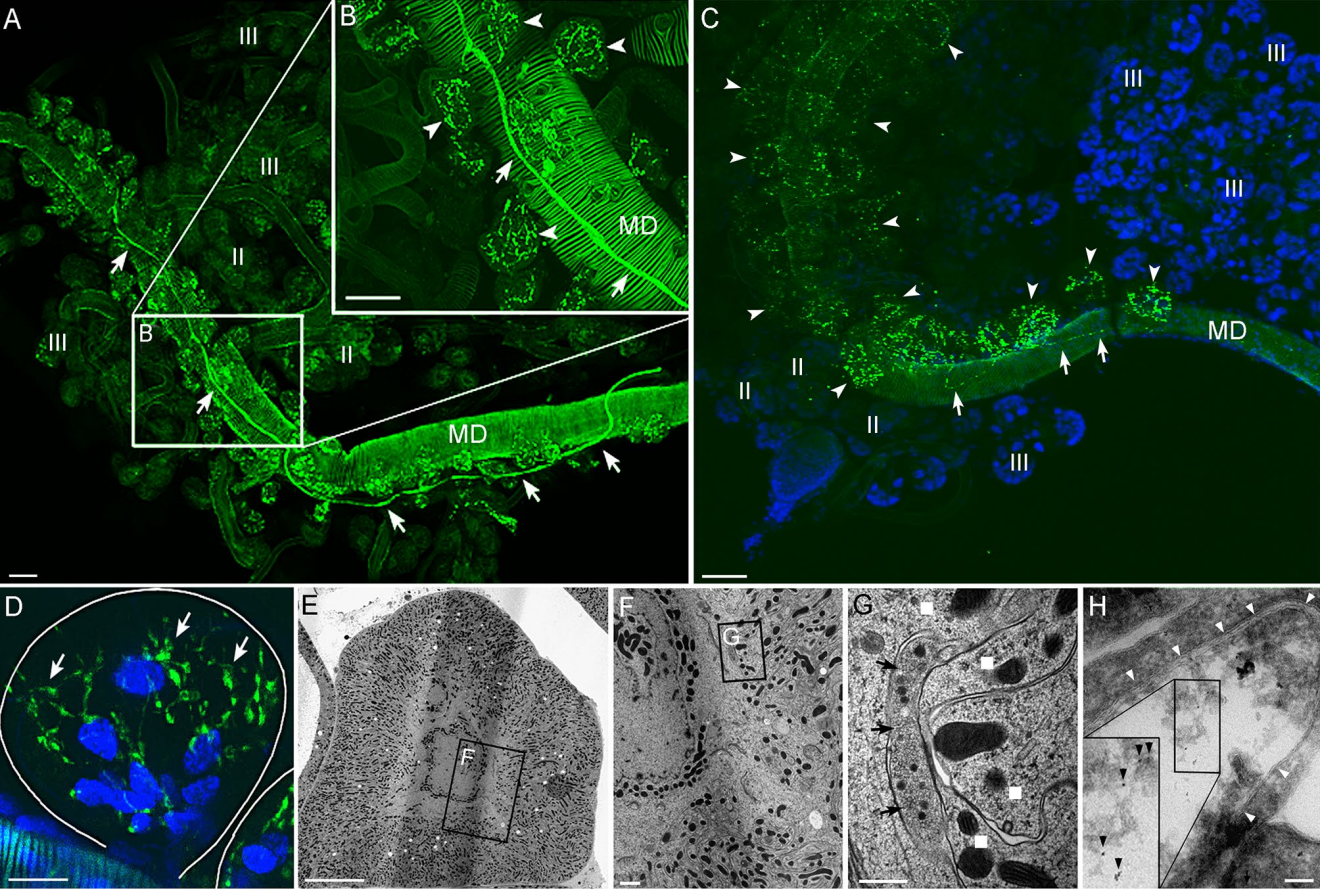
Immunostaining of ChAT in *Ixodes* salivary gland (SG). Whole-mount immunohistochemistry of *I. ricinus* (**A**,**B**) and *I. scapularis* (**C**) unfed female SG. Note that cholinergic axons (green) run along the main salivary duct (arrows) and terminate exclusively within the acini type I (arrowheads). Roman numbers II and III indicate type II and III acini located more distally from the main salivary duct (MD). (**D**) Detailed view of single type I acinus, highlighting its arborized cholinergic axon terminals (green, arrows). Solid lines indicate acinus boundaries. (**E**–**G**) Transmission electron microscopy (TEM) image showing the axons containing electron-dense vesicles (arrows in **G**) within the type I acinus in SG of five days fed *Ixodes* female. Insets in E and F are magnified in (**F**,**G**) respectively. Note that axons were found in contact with basolateral infoldings (white squares in **G**) of acinar lamellate cells. (**H**) TEM image showing immunogold ChAT antibody labeling of the axon within the type I acinus in SG of unfed female. The black arrowheads indicate the 6 nm gold nanoparticles within the axoplasm (inset), while the white arrowheads show the axolemma. Blue in **C** and **D** is DAPI staining for nuclei.  Scale bar is 50 μm in (**A**–**C**), 10 μm in (**D**, **E**), 1 μm in (**F**), 500 nm in (**G**), and 100 nm in (**H**).

A *D. melanogaster* antibody against ChAT identified several cholinergic neurons along their projections within the *Ixodes* synganglion (Fig. 2A). In the protocerebrum, reaction was recognized in six pairs of small protocerebral anterior-medial neurons (PcAM), and in two pairs of protocerebral dorso-lateral neurons (PcDL_1,2_). On the ventral side of the synganglion, a pair of postesophageal ventro-medial neuronal cells (PoVM) was located. ChAT was also strongly present in dense axonal network clusters on the ventral part of the opistosomal region. From the lateral aspects of these structures, thick axonal projections run posteriorly to enter the opistosomal nerves (OsN). While it was difficult to identify the origin of these axonal networks using IHC approaches, we successfully employed an anti-*chat* probe to visualize ChAT-encoding mRNA in neural somata. We used this approach to identify a pair of prominent opistosomal neurons (OsSG)—described as a source of SGs innervation—in the ventral part of the opistosomal ganglion (Fig. 2B). In addition, the same pair of neurons was also recognized by the anti-*vacht* probe (Fig. 2C). The combination of IHC and ISH on the same synganglion specimen revealed the connections between OsSG neurons and axons entering the OsN (Fig. 2D–F, Videos 1, 2) which subsequently innervate the type I acini (see below Fig. 3). In addition, the ISH procedure followed by IHC enabled the visualization of the segmental axonal processes exiting each of the pedal lobes I–IV (Fig. 2D–F).

### Cholinergic innervation of salivary glands

**Figure 4:**
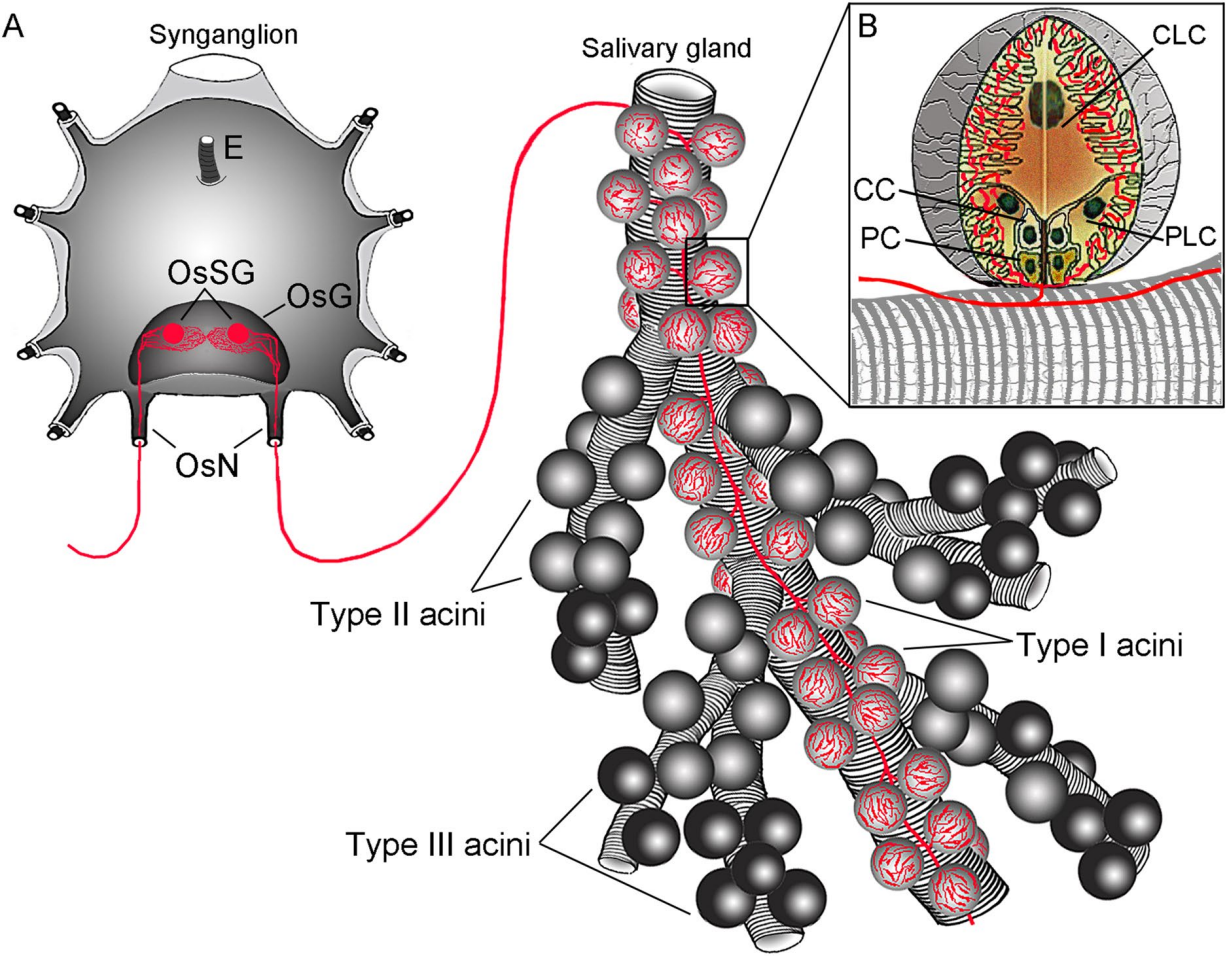
Schematic illustration of the cholinergic connection between the *Ixodes* synganglion and salivary gland. (**A**) The cholinergic axons (red) originating from OsSG neurons exit the synganglion via the opistosomal nerves (OsN) to reach the type I acini exclusively. (**B**) Schematic detailing the distribution of cholinergic axons (red) within the type I acinus. Note that cholinergic axon terminals within the acinus invaginate between the basolateral infoldings of both peripheral lamellate cells (PLC) and the central lamellate cell (CLC). *C* circumlumenal cell, *PC* peritubular cell, *E* esophagus, *OsG* opistosomal ganglion. The nomenclature of the acinar cells is after Needman et al.^26^.

Cholinergic OsSG neurons send their axons via the opistosomal nerves (OsN; Fig. 2A,D–F), and enter the anterior part of *Ixodes* SGs. In each individual SG, the single axon runs along the main salivary duct, and its short branches exclusively reach the type I acini (Figs. 3A–D, 4A, Video 3). The axon enters the individual acini via their neck regions, and arborizes within them into numerous axon terminals containing varicosities (Figs. 3D, 4B). Cholinergic axon terminals run close together at the basal part of the acinus, but remain further apart in the apical acinar region (Fig. 3D). Performing transmission electron microscopy (TEM) on an entire type I acinus highlighted axons containing several electron-dense neurosecretory vesicles (Fig. 3E–G). Specifically, an axon was found in close association with basolateral infoldings of a central lamellate cell (Fig. 3G). The TEM-immunogold labeling with anti-ChAT antibody confirmed this reaction within the type I acini axoplasm (Fig. 3H).

### Phylogeny and expression pattern of mAChRs

**Figure 5:**
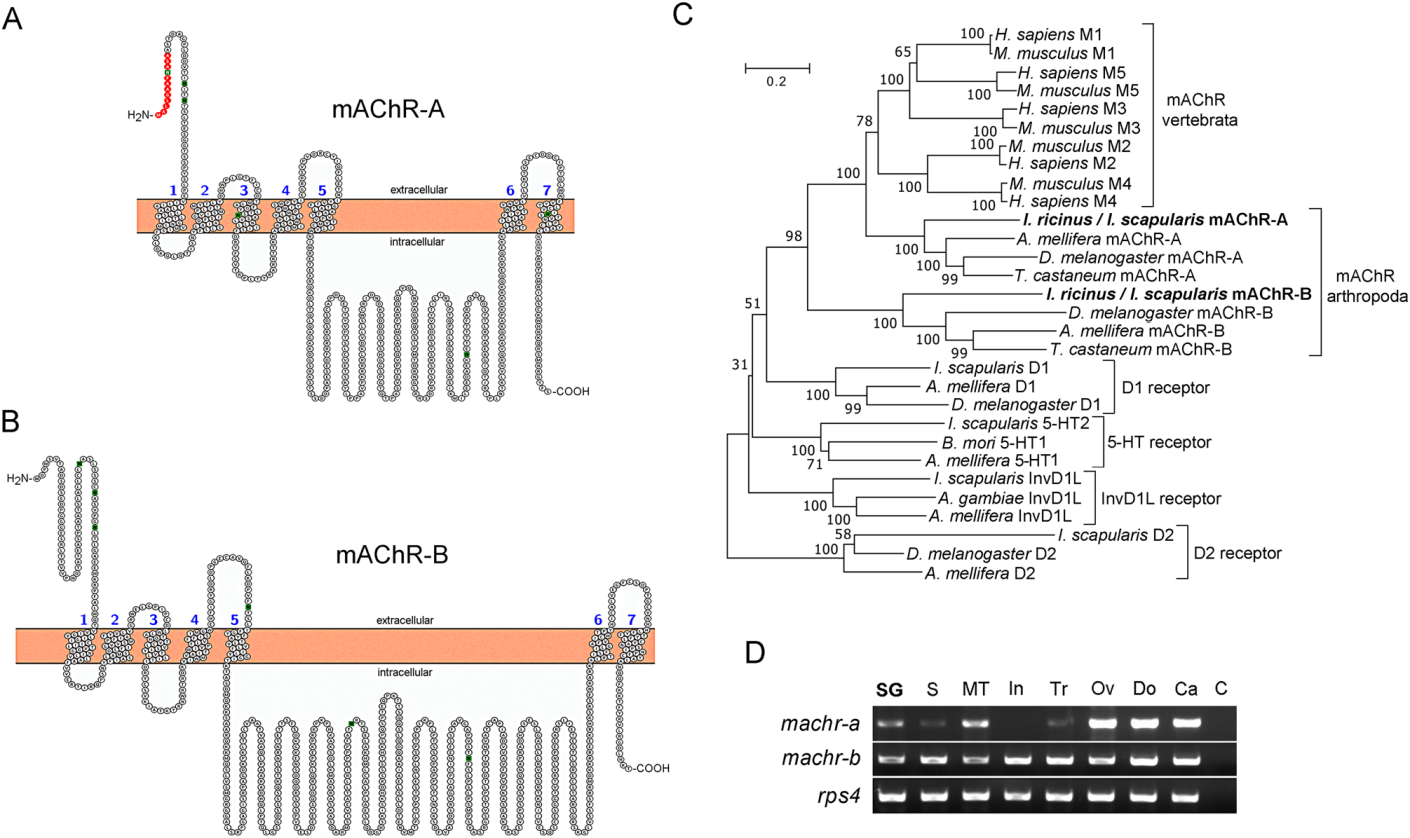
(**A**,**B**) Graphical transmembrane organization of mAChRs, using the Protter 1.0 software (https://wlab.ethz.ch/protter/start/). Red color in (**A**) indicates the signal peptide and green indicates the N-linked glycosylation motifs, blue numbers show transmembrane helices. (**C**) Phylogenetic relationship between vertebrate and arthropod mAChRs including mAChR-A and mAChR-B from *I. scapularis*/*I. ricinus*. *D1* D1 dopamine receptor, *InvD1L* invertebrate-specific D1-like dopamine receptor, *5-HT receptor* 5 hydroxytryptamine (serotonin) G-protein coupled receptor, *D2* D2 dopamine receptor. GenBank Accession numbers are: *Homo sapiens* mAChR M1, NP_000729.2; *Mus musculus* mAChR M1, NP_001106167.1; *H. sapiens* mAChR M5, NP_001307846.1; *M. musculus* mAChR M5, NP_991352.2; *H. sapiens* mAChR M3, NP_000731.1; *M. musculus* mAChR M3, NP_150372.1; *H. sapiens* mAChR M2, NP_000730.1; *M. musculus* mAChR M2, NP_987076.2; *M. musculus* mAChR M4, XP_006498717.1; *H. sapiens* M4, NP_000732.2; *I. scapularis*/*I. ricinus* mAChR-A, XM_002403091.2; *Apis mellifera* mAChR-A, XM_395760.7; *D. melanogaster* mAChR-A, JQ922421.1; *T. castaneum* mAChR-A, JX174094.1; *I. scapularis*/*I. ricinus* mAChR-B, XM_002416115.2; *D. melanogaster* mAChR-B, JX028235.1; *A. mellifera* mAChR-B, XM_006558358.3; *T. castaneum* mAChR-B, XM_015985417.1; *I. scapularis* D1, XM_029975666.1; *A. mellifera* D1, NM_001011595.1; *D. melanogaster* D1, NM_001260163.1; *I. scapularis* 5-HT2, EEC03313.1; *Bombyx mori* 5-HT1, KM236101.1; *A. mellifera* 5-HT1, NP_001164579.1; *I. scapularis* InvD1L, XM_002399612.2; *Anopheles gambiae* InvD1L, KU948225.1; *A. mellifera* InvD1L, NM_001011567.1; *I. scapularis* D2, XM_029969460.1; *A. mellifera* D2, NM_001014983.1; *D. melanogaster* D2, NM_001031909.2. The phylogenetic three in C was constructed using neighbor-joining method in MEGA7 software^41^, with 500 bootstrap replications. (**D**) Tissue-specific PCR of *machr-a* and *machr-b* in different tissues from partially-fed *I. ricinus* females. Full-length gels including amplicon sizes can be found in Supplementary Fig. S11. *rps4* ribosomal protein S4 transcript, *SGs* salivary glands, *S* synganglia, *MT* Malpighian tubules, *In* intestines (midguts), *Tr* tracheas, *Ov* ovaries, *Do* dorsa, *Ca* carcasses, *C* control (without template).

We experimentally identified the ORFs of both A and B mAChRs. *I. scapularis* and *I. ricinus* protein sequences of both mAChR types share 100% identity. The intron-less ORFs of mAChR-A and -B encode 580 and 826 amino acid residues respectively, and contain signatures for seven transmembrane domains, typical of G protein-coupled receptors (GPCRs) (Fig. 5A,B, see also Supplementary Figs. S6, S7). Phylogenetic analyses comparing vertebrate and arthropod mAChRs suggested clear orthologous clusters of *Ixodes* mAChR-A and mAChR-B in arthropod group of the mAChRs (Fig. 5C). Tissue specific RT-PCR of partially-fed *I. ricinus* females demonstrated *machr-a* expression in the SGs, synganglia, Malpighian tubules, tracheas, ovaries, dorsa, and carcasses, but not in the intestines, whereas *machr-b* was expressed in all examined tissues (Fig. 5D).

### Ligand-receptor interactions

**Figure 6:**
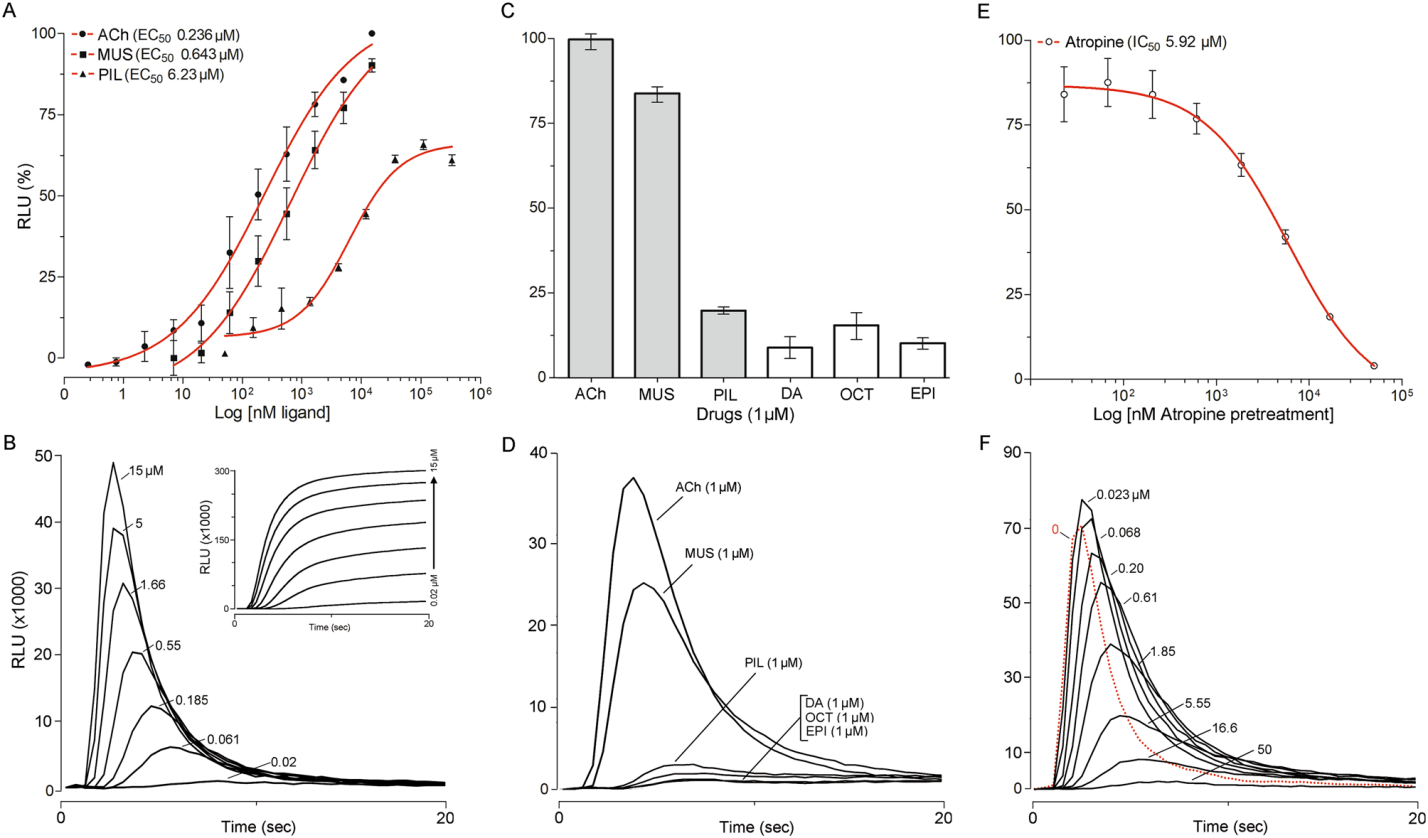
Bioluminescent aequorin reporter assays for mAChR-A expressed in CHO-K1 cells alongside the human G_α15(16)_ subunit. (**A**) Dose–response curves to various doses of acetylcholine (ACh), muscarine (MUS), and pilocarpine (PIL). (**B**) Representative 20-s typical luminescent responses to different doses of ligand (muscarine in this case). Inset in (**B**) shows the integrated relative luminescent values calculated from 40 intervals within the 20 s responses. (**C**) Luminescent responses to different drugs at 1 μM concentration. Dopamine (DA), octopamine (OCT), and epinephrine (EPI). (**D**) 20-s luminescent responses to 1 μM drugs. (**E**) Typical dose–response curve of antagonistic atropine activity. (**F**) Representative luminescent responses to 10 μM ACh after pre-incubation with different atropine concentrations. The bars in (**A**,**C**,**E**) indicate the standard error for three replicates. In (**A**,**E**) some standard error bars are smaller than the symbols used and in these cases, only the symbols are shown.

CHO-K1 cells expressing aequorin and G_α15(16)_ demonstrated significant mAChR-A activation responses to varying doses of different ligands (Fig. 6A–D). Specifically, among several drugs tested, ACh elicited the highest response, with an EC_50_ value of 0.236 μM, followed by muscarine with an EC_50_ value of 0.643 μM. Pilocarpine led to an approximately 25-fold-lower response with an EC_50_ value of 6.23 μM comparing to ACh (Fig. 6A). When cells were exposed to 1 μM ligand, muscarine generated ~ 85%, pilocarpine ~ 20%, dopamine ~ 8%, octopamine ~ 15%, and epinephrine ~ 10%, of the response of ACh (Fig. 6C,D). The mAChR-A antagonist atropine abolished ACh responses in a dose-dependent manner with an IC_50_ value of 5.92 μM (Fig. 6E,F). In CHO-K1 cells lacking the G_α15(16)_ subunit, ACh and muscarine also triggered calcium mobilization upon mAChR-A activation (see Supplementary Fig. S8). CHO-K1 cells transfected with only aequorin and G_α15(16)_ subunit constructs did not show any responses to ACh, muscarine, or pilocarpine.

mAChR-B expression in CHO-K1 cells either with, or without G_α15(16)_ subunits, followed by exposure to varying doses of ACh or muscarine, did not elicit any downstream calcium mobilization signal. Similarly, mAChR-B did not inhibit forskolin-mediated cAMP activity in HEK cells as assessed by the GloSensor reporter system (Supplementary Methods).

### Role of type I acini in water absorption by dehydrated ticks

**Figure 7:**
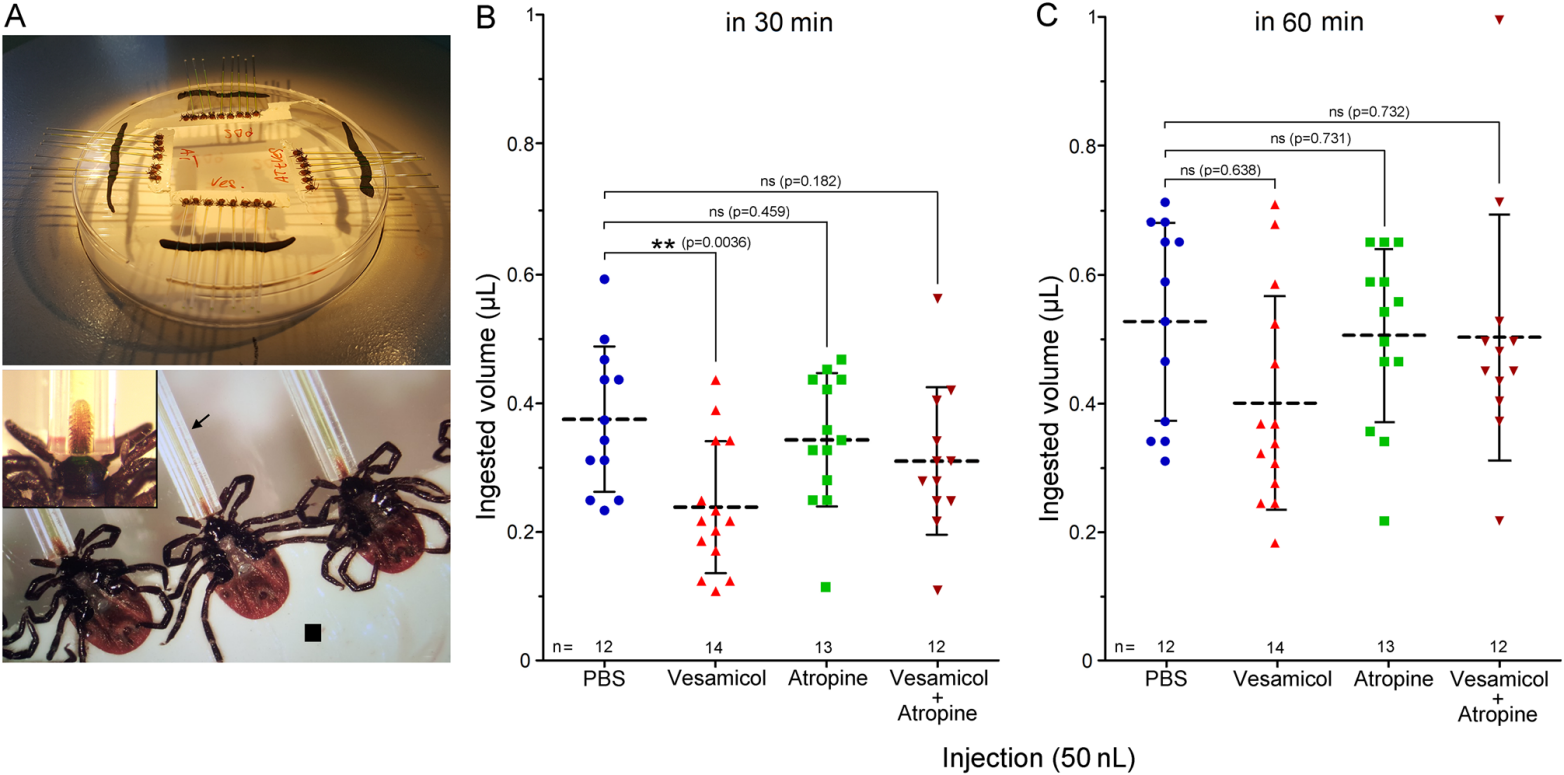
Effects of vesamicol and atropine on volume of water ingested by desiccated *I. ricinus* females. (**A**) Experimental set-up of forced water ingestion by desiccated ticks. Four different groups of *Ixodes* females (upper panel) were connected to glass microcapillary tubes (arrow, total volume 1 μl) via their hypostomes (lower panel). The black square shows the double-sided adhesive tape to which the ticks were attached upside-down. Inset in lower panel shows detail of the hypostome inserted into the glass microcapillary. (**B**) Ingested water volume by ticks pre-injected with PBS or different drugs at 30 min. (**C**) Ingested water volume by the same group of ticks as in (**B**) at 60 min. Each symbol in (**B**,**C**) indicates an individual *Ixodes* female. The horizontal dotted line represents the mean, and the bars the standard deviation. The experiment represents two biological replicates.

To examine the role of cholinergic axon terminals in type I acini, we tested the effect of atropine (an mAChR-A antagonist) and vesamicol (an inhibitor of VAChT) in a forced water absorption assay with dehydrated ticks (Fig. 7A). Specifically, we predicted that reducing synaptic release of ACh by vesamicol, and/or antagonizing the mAChR-A in SGs by atropine, may affect the absorptive activities of type I acini during forced drinking in dehydrated ticks. Indeed, ticks pre-injected with vesamicol ingested significantly less water in the first 30 min compared to the PBS-injected group, while ticks pre-injected with either atropine or an atropine/vesamicol mixture, did not show any differences in ingested volume (Fig. 7B). When left to ingest water for a further 30 min (60 min in total), the same groups of treated ticks did not show any significant differences in water volume absorbed compared to control group (Fig. 7C).

### Quantitative RT-PCR of *chat*, *vacht*, *machr-a*, and *machr-b* in ticks maintained under humid or desiccated conditions

**Figure 8:**
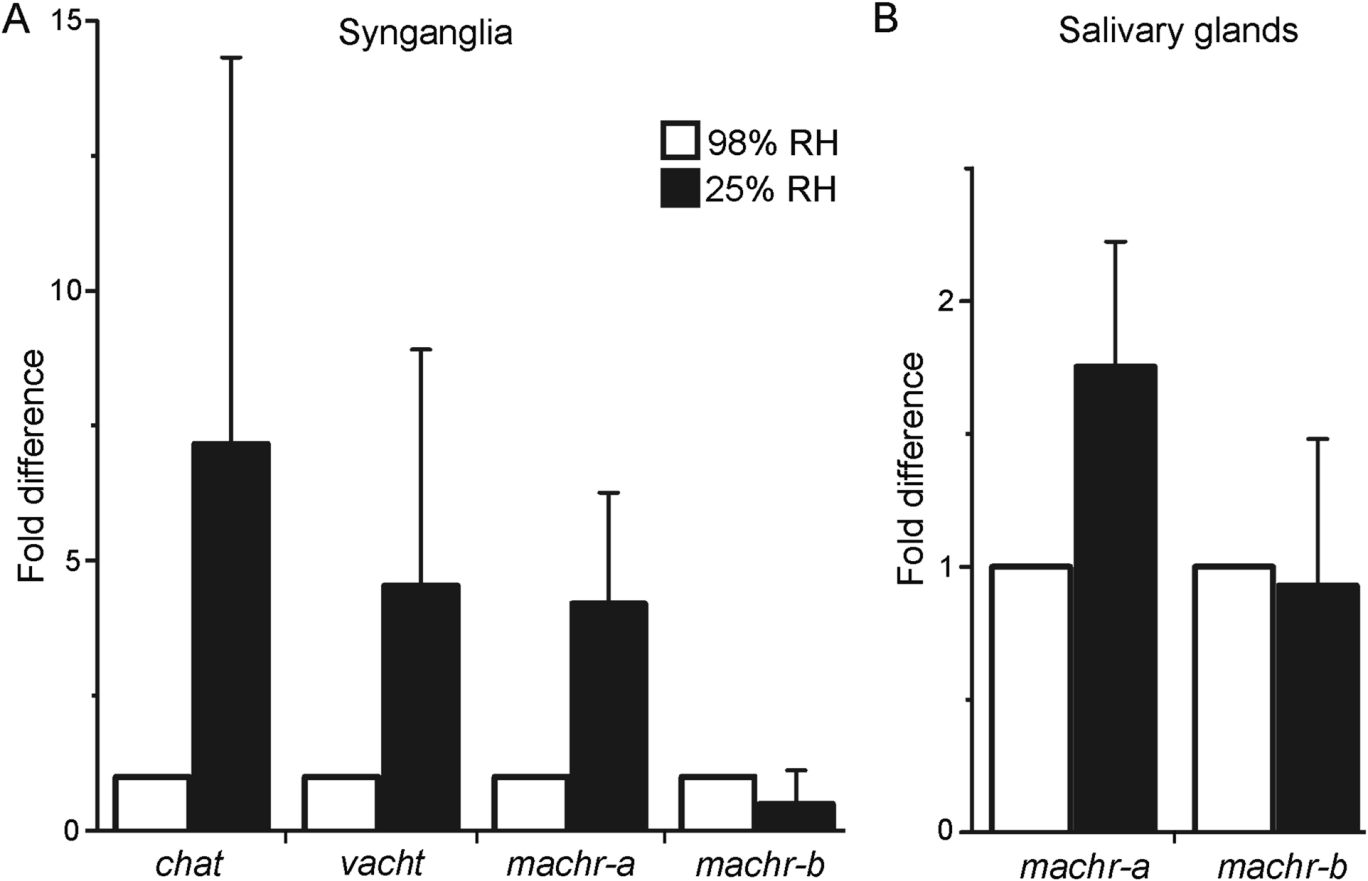
Quantitative RT-PCR showing the transcript levels of *chat*, *vacht*, *machr-a*, and *machr-b* in *I. ricinus* ticks maintained in 98% or 25% relative humidity (RH) for 30 h. (**A**) Transcript levels of *chat*, *vacht*, *machr-a* and *machr-b* in synganglia. (**B**) Transcript levels of *machr-a* and *machr-b* in SGs. Error bars indicate the standard error for two biological replicates. Data were normalized using the ribosomal protein S4 (*rps4*) transcript, and expression levels of specific transcripts from ticks maintained in 98% RH were assigned a value of 1. Note that comparing the mean to 1 for each transcript using a one-way Student t-test (P ≤ 0.05) did not show any statistically significant differences.

We investigated the variation in transcript levels of *chat*, *vacht*, *machr-a*, and *machr-b* in synganglia and, *machr-a* and *machr-b* in SGs, of unfed *Ixodes* females when ticks were exposed to severe dehydrating conditions (Fig. 8). The mean transcripts values of *chat*, *vacht*, and *machr-a* in tick synganglia (Fig. 8A), and *machr-a* in SGs (Fig. 8B)*,* appeared to be elevated in desiccated ticks. However, these differences were not deemed statistically significant, due to high individual variations between biological replicates. No statistically significant differences were observed in *machr-b* expression levels in either synganglia or SGs when comparing humid and desiccated conditions (Fig. 8A, B).

## Discussion

In arthropods, the ACh neurotransmitter is understood to be the primary excitatory compound at the synapse between neurons and their target cells^49,50^. Although the cholinergic system plays a vital role in tick physiology^33,51,52^, the knowledge of the specific processes involved in ACh synthesis, packing, and release, along the effects of cholinomimetic ligands at specific tick body sites remains largely obscure. Many groups have concentrated on characterizing tick acetylcholinesterases (AChE)—enzymes that catalyze ACh hydrolysis at the synapse—as they are potent targets for organophosphate acaricides^53–55^. An earlier study confirmed ChAT activity in synganglia extracts from *Rhipicephalus microplus*^56^, however, it is only in this current study that ChAT genomic organization and the full-length ChAT sequence has been characterized. The *Ixodes* cholinergic gene locus consists of two alternatively-spliced transcripts encoding ChAT and VAChT, in a configuration similar to that identified in other metazoans^57^. Interestingly, among all *chat/vacht* exons, only exon 4 of the *chat* gene appears to be located on an alternative scaffold of the *I. scapularis* genomic sequence. Although we cannot exclude a possible genome assembly error, the presence of transcripts omitting or including exon 4 (two and three transcripts, respectively) indicates a possible trans-splicing feature that has already been shown as an important regulatory factor of mRNA processing in insects^58–60^. The high amino acid identity of ChAT and VAChT protein sequences between *I. ricinus* and *I. scapularis* support their close evolutionary relationship, and suggests a common physiological role for the cholinergic system in these two allopatric hard tick species.

IHC staining of the ChAT protein revealed various types of neurons and their projections within the *Ixodes* synganglion, including prominent pairs of neurons (OsSG) identified as a source of the cholinergic innervation of *Ixodes* SGs (see below). Interestingly, ISH approaches confirmed the exclusive presence of both *chat* and *vacht* mRNA in the somata of OsSG neurons. The discrepancies in the number of neurons visualized by these two techniques can be explained by (i) nonspecific cross-reactivity of *Drosophila* anti-ChAT antibody with unknown protein(s) in some *Ixodes* neuronal cells, or (ii) undetectable *chat* and *vacht* mRNA levels in some cholinergic neurons. In either case, both IHC (for ChAT) and ISH (for *chat* and *vacht*) identified the single pair of opistosomal neurons as the origin of type I acini innervation in *Ixodes* SGs. In addition, we learnt that performing ISH followed by IHC on synganglia dramatically enhanced immuno-detection in the neurons and their projections within this tissue, possibly as a by-product of enhancing membranes permeabilization during the first procedure. Despite the fact that the ultrastructure of tick SG has been well described, information regarding the axons innervating type I SG acini remains elusive^26^. Based on confocal and TEM approaches, we showed that the highly abundant cholinergic axon terminals in type I acini may target basolateral infoldings of two different cell types: (i) peripheral lamellate cells that are the first cells in contact with these axons entering the acinus and/or (ii) the single central lamellate cell in contact with central and apical parts of axon terminals.

The mAChR has been suggested to play a crucial role in tick SG physiology since the cholinomimetic agent, pilocarpine, was the first pharmacological compound found to stimulate tick SG secretion in vivo^61,62^. The actions of cholinomimetic drugs have been tested across several species in the ixodid family^16,63,64^, and current models suggest that pilocarpine-mediated salivation is linked to putative mAChR activation in tick synganglion, that subsequently stimulates an unidentified secreto-motor nerve directly innervating the SG^33^. In addition, our recent study also showed that pilocarpine induces tick chelicera movement, suggesting that this drug may have a complex effect on *Ixodes* feeding behaviour^65^. The hypothesis that mAChR may be a cholinoceptive site for salivation has also been confirmed by effectively blocking pilocarpine-mediated fluid secretion with atropine, a typical mAChR-A antagonist^33^. Here, we identified two types of mAChR (A and B), both expressed in *Ixodes* synganglion and SG. The *Ixodes* mAChR-A thought to be linked to the G_q/11_ pathway showed high biological affinity to the atropine blocker in our experiments, as has been also reported in *Drosophila*^66^. Interestingly, sensitivity to pilocarpine agonist, had approximately 25 × lower activity for this drug compared to ACh, indicating that pilocarpine is a non-potent activator of the receptor. These data also correlate with studies of mammalian mAChR-A orthologues, where pilocarpine also had low biological affinity to the receptor^67,68^. On the other hand, the potent endogenous mAChR-A agonist ACh, failed to effectively stimulate secretion in ixodid ticks^33^, likely due to an inability to permeate tick tissue barriers, whereas pilocarpine has a documented high penetration ability^64^. Although two heterologous systems directly monitoring either calcium or cAMP downstream signals were used, we were unable to detect activation of type B mAChR. In *Drosophila,* mAChR-B is thought to be linked to the G_αi/o_ pathway inhibiting cAMP production, and does not appear to be sensitive to atropine blockers^66^. In contrast, the orthologous mammalian M2 muscarinic receptor is known to be coupled to G_β_/G_γ_ subunits in heartbeat regulation, thus directly activating the G protein-activated inward rectifier potassium channel GIRK^69^. Unsuccessful functional expression studies of *Ixodes* mAChR-B are likely due to downstream receptor incompatibility in our expression system. Thus, taken together, more thorough investigations are required to conclude whether pilocarpine-mediated SG fluid secretion is regulated via mAChR(s) or other system(s).

Despite lacking direct experimental proof, it has been generally believed that type I acini in hard ticks are the source of hygroscopic saliva forming humid-binding crystals onto their hypostome surface^70–72^. Just recently, two studies proved that hygroscopic saliva is produced by type II/III acini, while ion and water absorptive functions were suggested to be exclusively due to type I^25,32^. In desiccated ticks, ingested water coming from the deliquesced hygroscopic crystals is absorbed via an electrochemical gradient created by Na^+^/K^+^-ATPase located on basolateral infoldings of lamellate cells in type I acini^25,32^. In this article, cholinergic axon terminals reaching the same regions of lamellate cells were described and we predicted that stimulation of putative postsynaptic mAChR, triggers Ca^++^-mediated activation of protein kinase C, leading to activation of transporters (i.e. Na^+^/K^+^-ATPase and possibly V-ATPase) for resorption of Na^+^ and water in acini type I. Similar mechanism has been described in cockroach SG^73^. Therefore, we designed an experiment to test whether disrupting synaptic ACh release and/or blocking postsynaptic mAChR in type I acini may affect water ingestion in severely dehydrated ticks. Although we observed substantial variation in the amount of ingested water between individual *Ixodes* females, significantly less volume was ingested by ticks treated with vesamicol, a drug inhibiting ACh uptake by synaptic vesicles and thus reducing its release into the synapse^74^. Interestingly, ticks treated with the mAChR-A antagonist, atropine, ingested approximately the same volume of water as control ticks. Similarly, no effect on ingestion volume was observed in ticks treated with the vesamicol/atropine mixture. Here, we question whether the drugs injected into the haemocoel effectively reached their cognate transmembrane proteins in SG, or if the pre-incubation time was sufficient for their maximum efficacy, or if the drugs remained stable during the entire experiment. This assumption is supported by the fact that in our assay, vesamicol effects were evident within the first 30 min, which then started to slow over time. Moreover, the absence of measurable effects from atropine, and possibly from atropine/vesamicol, could be explained by the muscarinic receptor(s) affinity inhibition by Na^+^/K^+^-ATPase activity in type I acini, a mechanism that has previously been described in invertebrates^75^. We also cannot exclude the possibility of the mAChR role as an autoreceptor, where the presynaptic mAChR(s) is under the feedback control like the cases shown in insect^76^. Furthermore, systemic effects of injected drugs should be taken into account, since fluid ingestion by ticks involves several primary tick feeding apparatus^65^. In addition we were interested to see if cholinergic synapses within type I acini are active during tick desiccation. Surprisingly, due to large individual variation, we didn’t observe statistically significant differences in cholinergic transcript expressions between desiccated and control ticks in either tick synganglia (*chat*, *vacht*, *machr-a*, and *-b*) or SGs (*machr-a* and -*b*). In either case, our data indicate that one of the roles of cholinergic axons in *Ixodes* SG may be to regulate lamellate cell activity in type I acini during the off-host period.

Barker et al.^77^ observed that during tick feeding, the mitochondrial dissolution, lipid coalescence and depletion, as well as autophagic structure accumulation in certain type I acini cells, all suggest an important role for type I acini during the on-host period. In addition, a recent study described the important resorptive functions of Na^+^/K^+^ pump in forming isosmotic saliva in type I acini during tick feeding^32^. Therefore, we predicted that knocking down elements of the cholinergic synapse in *Ixodes* nymphs may disrupt SGs functionality (and possibly other organs), hindering the feeding and subsequent molting. Neither knock-down of *chat*, *vacht*, *machr-a*, nor *machr-b* genes influenced tick bloodmeal uptake, feeding duration, or molting of nymphs into adults. In addition, only silencing *chat* in synganglia (64.4% knock-down expression compared to control) appeared to be significant, while other tested genes did not show any notable silencing effects after dsRNA injection (Supplementary Figs. S9, S10, Supplementary Methods). Unsuccessful attempts in gene silencing in our experiments are likely due the unknown molecular factors in specific tissue, limiting the silencing efficacy. Thus, more studies are required to understand the obstacles in inefficient or highly variable results of RNA interference in ticks research.

In the current study we investigated the cholinergic pathways in *Ixodes* SG. Our results suggest, that activities of cholinergic synapse in type I acini may play a role in water absorption by desiccated ticks. Although this finding does not replicate previous reports suggesting indirect cholinergic control of secretion activities of tick SGs via the synganglion^8,33,78^, it can’t be excluded. Currently, the only candidates that could directly connect the synganglion with saliva-producing type II and III acini are the neuropeptidergic axons^19–21,23,24^, while the origin of InvD1L-expressing axons innervating the same acini types remains to be identified^22^. Whether some of these neurons express cholinergic receptor(s) sensitive to pilocarpine, and thus indirectly activate SG secretion remains poorly understood. Our ongoing research (a current project in Šimo’s laboratory) will place additional focus on these aspects. These studies would further establish the localization of the expressed mAChRs proteins in tick synganglion, SG, and possibly other organs, as the mRNA encoding these receptors appears to be present throughout several tick tissues.

The newly revealed innervation of type I acini in our study has filled a missing knowledge gap, and is important in understanding the complex nature of neural mechanisms regulating SGs in *Ixodes* ticks.

## Supplementary information

Supplementary Information 1.

Supplementary Information 2.

Supplementary Video 1.

Supplementary Video 2.

Supplementary Video 3.

## Data Availability

All data generated or analyzed during this study are included in this published article (and its Supplementary Information files).
