# Supplementary material for: Cholinergic axons regulate type I acini in salivary glands of Ixodes ricinus and Ixodes scapularis ticks
Source: Sci Rep. 2020 Sep 29;10:16054. doi: 10.1038/s41598-020-73077-1 (PMC7524744; doi:10.1038/s41598-020-73077-1)
Supplement: Supplementary file 1 — Supplementary Information 1. [file 41598_2020_73077_MOESM1_ESM.docx]

Supplementary information

Cholinergic axons regulate type I acini in salivary glands of *Ixodes ricinus* and *Ixodes scapularis* ticks

Lourdes Mateos-Hernandéz^1†^, Baptiste Defaye^1,2†,§^, Marie Vancová^3,4^, Ondrej Hajdusek^3^, Radek Sima^3^, Yoonseong Park^6^, Houssam Attoui^7^ and Ladislav Šimo^1*^

^1^UMR BIPAR, INRAE, Ecole Nationale Vétérinaire d’Alfort, ANSES, Université Paris-Est, Maisons-Alfort, France

^2^Université de Limoges, Faculté de Pharmacie, Limoges, France

^3^Biology Centre, Institute of Parasitology, Czech Academy of Sciences, Ceske Budejovice, Czech Republic

^4^Faculty of Science, University of South Bohemia, Ceske Budejovice, Czech Republic

^6^Department of Entomology, Kansas State University, 123 Waters Hall, Manhattan, KS, USA

^7^UMR Virologie, INRAE, Ecole Nationale Vétérinaire d’Alfort, ANSES, Université Paris-Est, Maisons-Alfort,

France

† Equal contribution

§ Current address: UMR SPE 6134 CNRS, Université de Corte Pascal Paoli, Corse

* Corresponding author: ladislav.simo@vet-alfort.fr

**Name Forward primer (5’ – 3’) Reverse primer (5’ – 3’)**

***In situ* hybridization:**

*Chat (exons 9-14)* ATGCAGATAGTCGTCAGCCG TCAACTCGCTGTGGGAGGAA

*Vacht(exon 2)* TTGGTGTGCTGTTCGCCTCC ATGTAAGGATCGATGAAGAG

**ORF cloning:**

*machr-b* TGCAGGTGTGCTTCCTGAGGCA TATGCCGCAACTAACGGTTA

*machr-a* TGACCAGAGGCACAAGTCATG AGCGGCTCAACTGAAGTAA

**Tissue-specific PCR:**

*machr-a* TGATAAGGAGGACGACGACA AGCACGAGAACGTTGTACGG

*machr-b* TGCAGGTGTGCTTCCTGAGGCA AGCACGTAGAAGGCGTAGAA

*nachr sub.ß* TTGTCCTTCATCCAGCTCAT TCGTTATACAGCTTTAAGGA

***chat* and *vacht* exons:**

*shared exon-vacht*  GACAGCCCCAGACGAAAGCT ATGGTAAGACCAACCATCAT

*chat(exons 12-14)* AAGCTGGTGTCGACGTACG GCTTGGAAAAACTTGATCCT

*chat(exons 13-15)* AGATGGCCAGGCTACACGAA AAGAAAGAGGACACGCAGAA

**qRT-PCR:**

*chat* ATGCAGATAGTCGTCAGCCG GACTTGCTGGCTTCCTGGAT

*vacht* GGAAGGTAGGCTTGAAGG   CACAAGCAGCAACGAACGAA

*machr-a* ACGAGACGACCACCACTTTC GTCACACGAGGAGACGGTTT
*machr-b* AAGCACGACAGGTCTTCCAG GAAGGCAATGACGGGAGTGA

**Supplementary Table S1.** Primers used in this study.

**
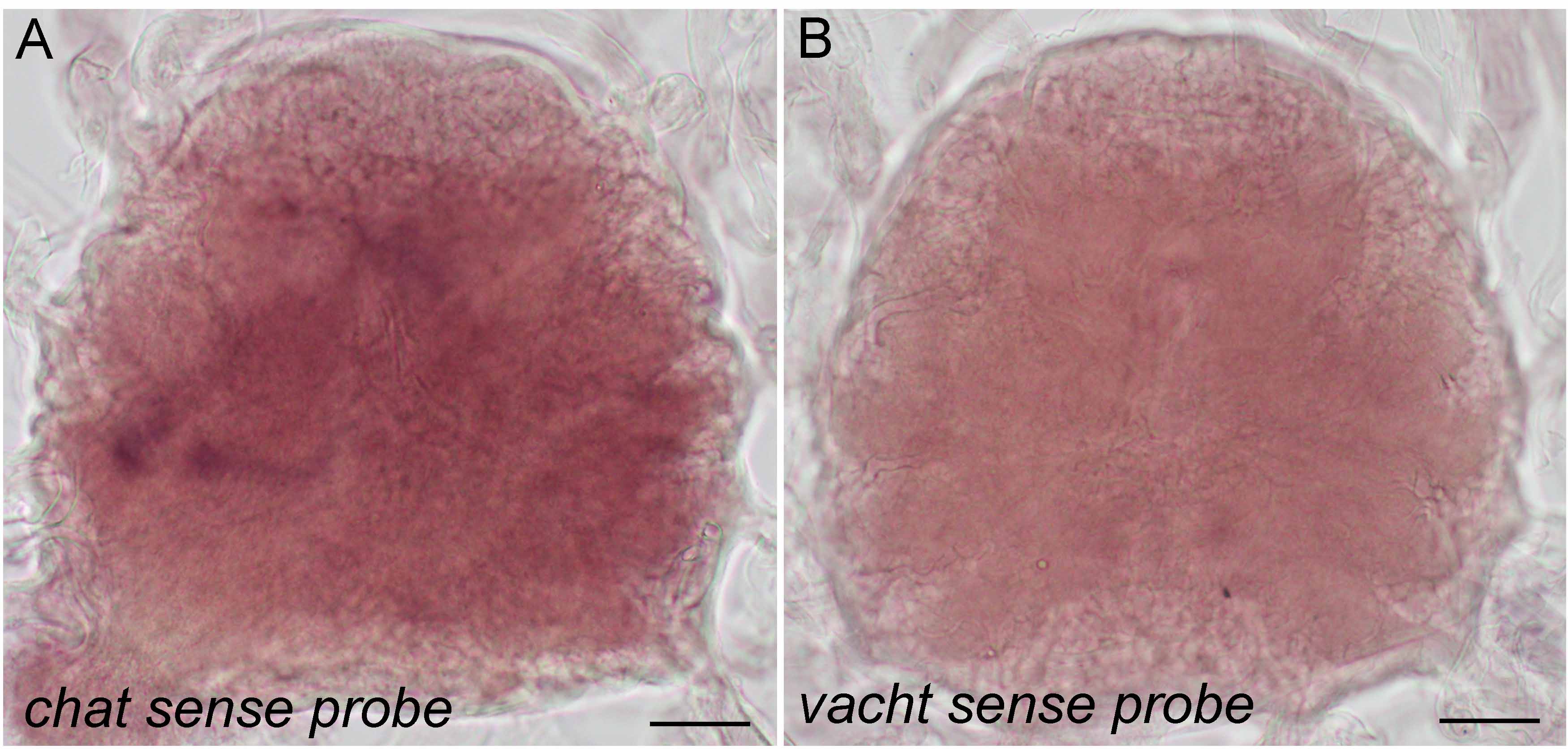
**

**Supplementary Figure S1.** Negative control of *chat* and *vacht* *in situ* hybridization. (A) Wholemount synganglion treated with *chat* sense probe. (B) Wholemount synganglion treated with *vacht* sense probe. Note that hybridization with sense probes did not show any reaction for both targets in *Ixodes* synganglia. Scale bars are 50 μm.

XM_029980779.1 ------------------------------------------------------------ 0

ISCW022171-RA MEVPCRVPSSIPRVRVLGCGPEAGNSRESRISRRLGLPTMDNRKCAPAYVIFRVHRDLQA 60

ISCI022171-RA MEVPCRVPSSIPRVRVLGCCPEAGNSRESRISRRLGLPTMGDRKCAPAYVIFRVHRDSQA 60

GBBN01014222.1 ------------------------------------------------------ATSQKA 6

XM_029980779.1 ------------------------------------------------------------ 0

ISCW022171-RA CDELLRAPVPALATTLQRYVDSLVAVVSPAQLEATRRIVRDFAGSSAEQGPPRDDGEGAA 120

ISCI022171-RA CDELLRAPVPALATTLQRYVDSLVAVVSPAQLEATRRIVRDFAGSSAEQGPPRDDGEGAA 120

GBBN01014222.1 CDELLRAPVPALATTLQRYLDSLVAVVSPAQLEATRRIVRDFAGSSAEQGPPRDEGEGAA 66

XM_029980779.1 ---------------------------------------MYLNNDQPLPVNSSPFCLLPK 21

ISCW022171-RA SAPAAGATKTAPLGPLLQEKLRLFASSRDNWVC--------------------------- 153

ISCI022171-RA SAPAAGATKTAPLGPLLQEKLRLFASSRDNWVC--------------------------- 153

GBBN01014222.1 SAP--GATKTAPLGPLLQEKLRLFASSRDNWVTELWLDDMYLNNDQPLPVNSSPFCLLPK 124

XM_029980779.1 QNFRTSAEQARFAARFIEFAVLFKRKIDNGTLRPDVSRSRGGGQQPLCMQTYRHFFPAYR 81

ISCW022171-RA ----------MFAARFIEFAVLFKRKIDNGTLRPDVSRSRGGGQQPLCMQTYRHFFPAYR 203

ISCI022171-RA ----------MFAARFIEFAVLFKRKIDNGTLRPDVSRSRGGGQQPLCMQTYRHFFPAYR 203

GBBN01014222.1 QNFRTSAEQARFAARFIEFAVLFKRKIDNGTLRPDVSRSRGGGQQPLCMQTYRHFFPAYR 184

*************************************************

XM_029980779.1 RPGPNKDDLLLDTAMQQQDHVIVACRDQFFRLKLPLDKEELDTEAIVEQLLSIKRRAKDP 141

ISCW022171-RA RPGPNKDDLLLDTAMQQQDHVIVVCRDQ---------------------LLSIKRRAKDP 242

ISCI022171-RA RPGPNKDDLLLDTAMQQQDHVIVACRDQ---------------------LLSIKRRAKDP 242

GBBN01014222.1 RPGPNKDDLLLDTAMQQQDHVIVVCRDQFFRLKLPLDNEELDTEAIVEQLLSIKRRAKDP 244

***********************.**** ***********

XM_029980779.1 SERQPPVGILTTENRRTWSNLYVKLSRSNVNQCSLQSLETCLLVVCLDRPLNLRRHYATI 201

ISCW022171-RA SERQPPVGILTTENRRTWSNLYVKLSRSNVNQCSLQSLETCLLVVCLDRPLNLRRHYATI 302

ISCI022171-RA SERQPPVGILTTENRRTWSNLYVKLSRSNVNQCSLQSLETCLLVVCLDRPLNLRRHYATI 302

GBBN01014222.1 SERQPPVGILTTENRRTWSNLYVKLSRSNVNQCSLQSLETCLLVVCLDRPLNLRRHYATI 304

************************************************************

XM_029980779.1 RRESFTLDWAAQAAHLLHGESNTPGEGNAANRWYDKFMQ----AIVSRDGVNGLIIEHSG 257

ISCW022171-RA RRESFTLDWAAQAAHLLHGESNTPGEGNAANRWYDKFMQASIDIVVSRDGVNGLIIEHSG 362

ISCI022171-RA RRESFTLDWAAQAAHLLHGESNTPGEGNAANRWYDKFMQASIDIVVSRDGVNGLIIEHSG 362

GBBN01014222.1 RRESFTLDWAAQAAHLLHGESNTPGEGNAANRWYDKFMQ----IVVSRDGVNGLIIEHSG 360

*************************************** :***************

XM_029980779.1 SDGVTVLRFCEEFLDFVQEHSVCSSARRGSGDTSLYPVSRLSWDLNEDMLRAIQEASKSL 317

ISCW022171-RA SDGVTVLRFCEEFLDFVQEHSVCSSARRESGDTSLYPVSRLSWDLNEDMLKAIQEASKSL 422

ISCI022171-RA SDGVTVLRFCEEFLDFVQEHSVCSSARRGSGDTSLYPVSRLSWDLNEDMLRAIQEASKSL 422

GBBN01014222.1 SDGVTVLRFCEEFLDFVQEHSVCSSARRGSGDTSLYPVSRLSWDLNEDMLRAIQEASKSL 420

**************************** *********************:*********

XM_029980779.1 GKLAEDVDLYVLTFQNYGKDFIKAQKISPDVFIQLALQLTYYKVHRKLVSTYESASLRKF 377

ISCW022171-RA GK-----------------------------------------VHRKLVSTYESASLRKF 441

ISCI022171-RA GK-----------------------------------------VHRKLVSTYESASLRKF 441

GBBN01014222.1 GKLAEDVDLYVLTFQNYGKDFIKAQKISPDVFIQLALQLTYYKVHRKLVSTYESASLRKF 480

** *****************

XM_029980779.1 HLGRVDNIRAATSEALTWIQAMCDAVPAS**EDDRIKFFQAAVNKGTEILTY**TVNGEGPDNH 437

ISCW022171-RA HLGRVDNIRAATSEALTWIQAMCDAVPAS---------------------TVNGEGPDNH 480

ISCI022171-RA HLGRVDNIRAATSEALTWIQAMCDAVPAS------------------------------- 470

GBBN01014222.1 HLGRVDNIRAATSEALTWIQAMCDAVPAS**EDDRIKFFQAAVNKGTEILTY**TVNG------ 534

*****************************

XM_029980779.1 LLGLREMARLHEKTVPLFEDKSYADFLRFRLSTSQ---------------------LATE 476

ISCW022171-RA LLGLREMARLHEKTVPLFEDKSYADFLRFRLSTSQ**EDDRIKFFQAAVNKGTEILTY**LATE 540

ISCI022171-RA -----------------------------------**EDDRIKFFQAAVNKGTEILTY**LATE 495

GBBN01014222.1 ------------------------------------------------------------ 534

XM_029980779.1 KSILVGYGPVVPDGYGCSYNICPAHVDFCVSSFFSSPETSSDFFALSLEGSLLQMRELCI 536

ISCW022171-RA KSILVGYGPVVPDGYGCSYNICPAHVDFCVSSFFSSPETSSDFFALSLEGSLLQMRELCI 600

ISCI022171-RA KSILVGYGPVVPDGYGCSYNICPAHVDFCVSSFFSSPETSSDFFALSLEGSLLQMRELCI 555

GBBN01014222.1 ------------------------------------------------------------ 534

XM_029980779.1 KRAQKDAENNAVLPPTAS 554

ISCW022171-RA KRAQKDAENNAVLPPTAS 618

ISCI022171-RA KRAQKDAENNAVLPPTAS 573

GBBN01014222.1 ------------------ 534

**Supplementary Figure S2.** Alignment of conceptual translations for the putative ORF of ChAT-related sequences found in *I. scapularis* transcript databases (NCBI and Vectorbase). Asterisks and dots indicate identical and similar amino acids respectively. Note that all sequences were computationally predicted from genome sequence. The letters highlighted in yellow represent the amino acid sequence encoded by exon 4 considered to be a putative trans-spliced exon. Amino acids sequences encoded by mutually-exclusive exons 14 (red letters) and 13 (red background) are also shown (see Suppl. Fig. S3). For all exon boundaries see Suppl. Fig. S4.


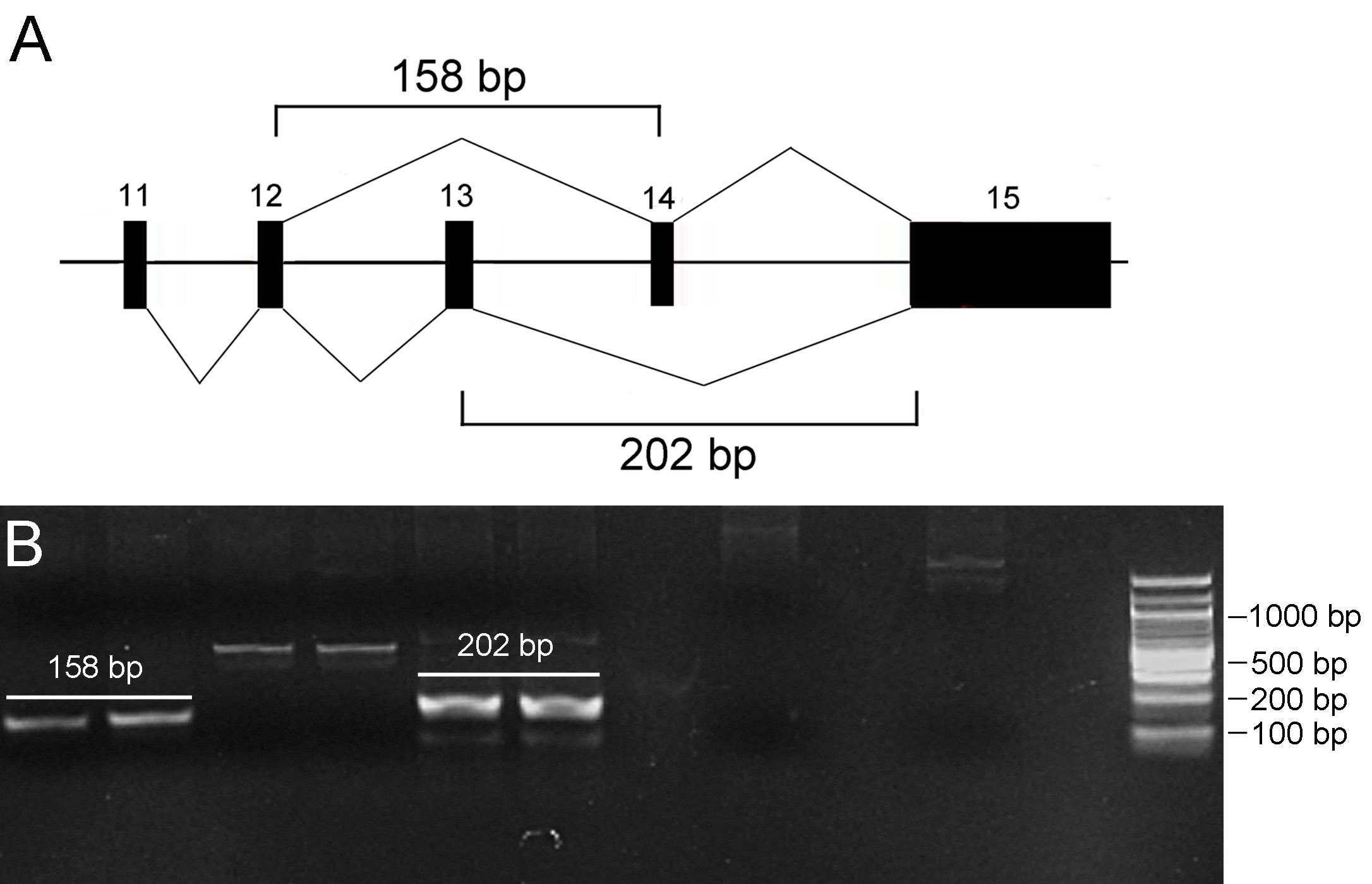


**Supplementary Figure S3.** Experimental confirmation of the mutually-exclusive exons 13 and 14 of the gene encoding ChAT. (A) Detail of the examined region (scaffold DS9106530, see Fig. 1A in the main text), of the mutually-exclusive spliced transcript variants. Horizontal lines represent introns, while vertical lines or boxes represent exons (numbered) and splicing between them is indicated with diagonal lines. (B) Full-length gel picture of RT-PCR products of the amplicon (two wells for each) at 158 bp (specific primers for exon 12 and 14) and 202 bp (specific primers for exons 13 and 15). For primer details see Supplementary Table S1.


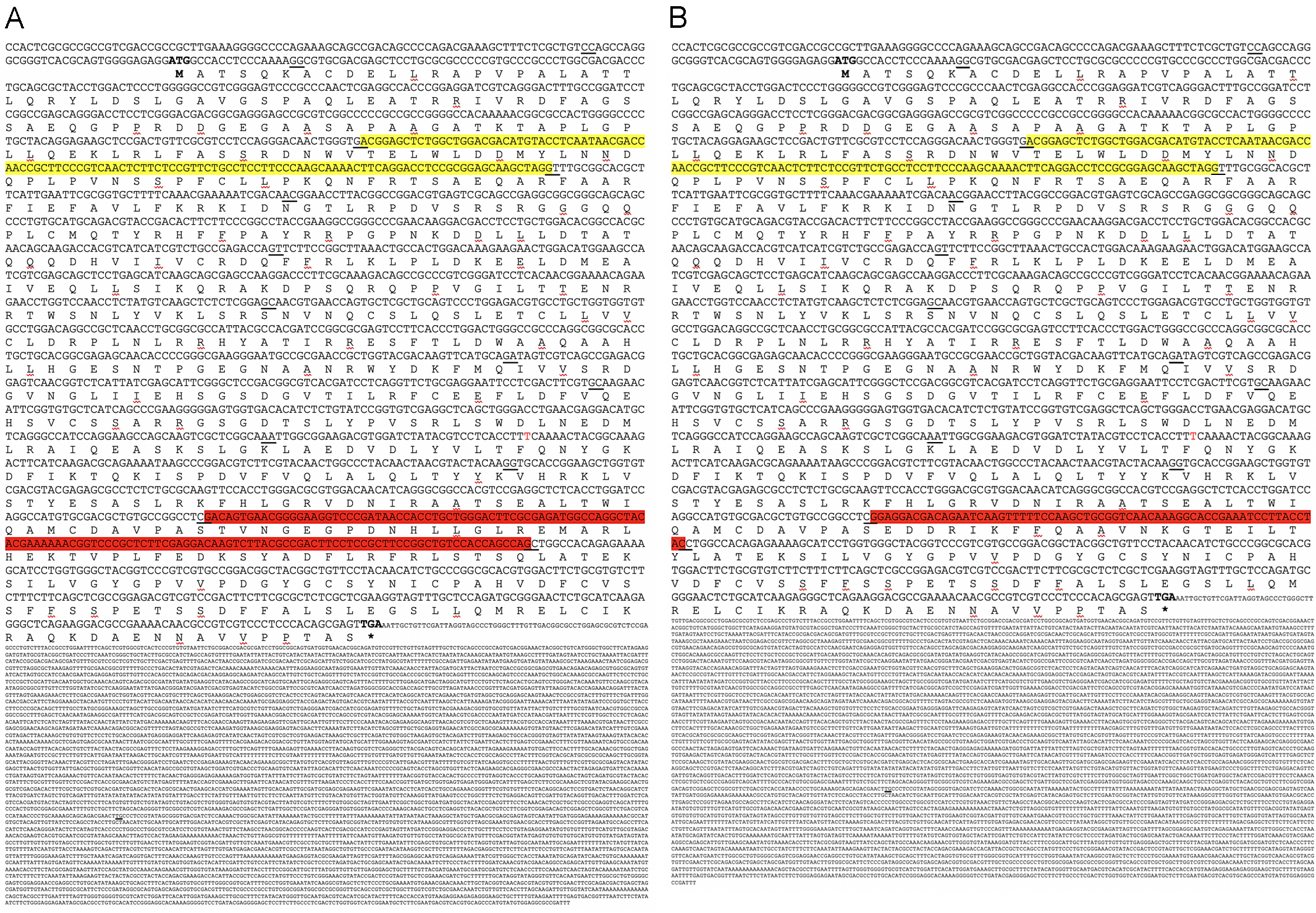


**Supplementary Figure S4.** Nucleotide sequence and conceptual translations of the cDNA encoding ChAT isoforms A (A) and B (B) in *I. ricinus*. The nucleotide sequences at the exon-intron junctions are underscored. The translation initiation signal and stop codon (asterisk) are in bold letters. The letters highlighted in yellow indicate the putative trans-spliced exon (number 4, also see Fig. 1. in main text), and letters highlighted in red indicate the mutually-exclusive exons 13 (A) and 14 (B), also see the Suppl. Fig. S3. and Fig. 1. in the main text. Transcript variant sequences were submitted to GenBank with accession numbers for *I. ricinus* ChAT isoform A and B (MT669641, MT669642 respectively). GenBank accession numbers for *I. scapularis* ChAT isoform A nad B are MT669643, MT669646, respectively.

**
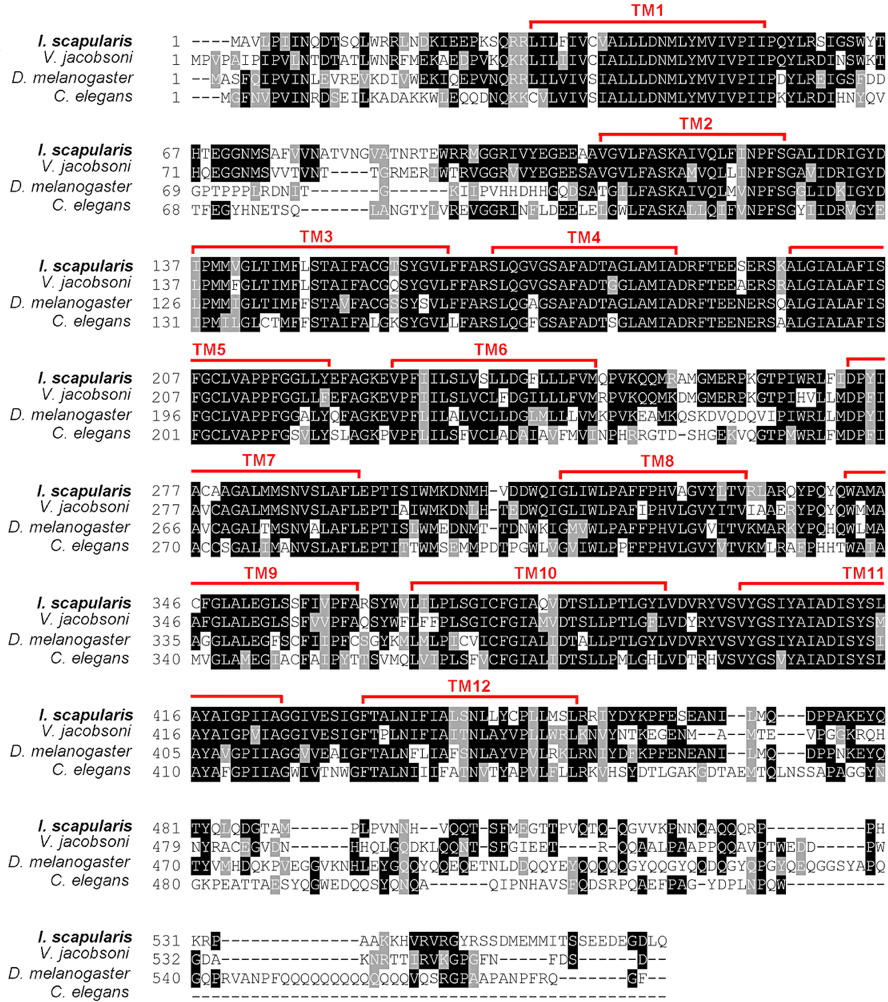
**

**Supplementary Figure S5.** Alignment of conceptual translations for VAChT-related sequences. Letters with gray background are similar amino acids, and letters with black background are identical amino acids in the sequence alignment using 50% majority rules. Seven conserved transmembrane segments of *Ixodes* VAChT are marked with red brackets above the alignment. The prediction of transmembrane segments was performed using TOPCONS software. For GenBank accession numbers for *I. scapularis*, *V. jacobsoni*, and *D. melanogaster* VAChT, see Fig. 1 caption in the main text. The *C. elegans* VAChT GenBank accession number is NM_001028431.4.

**
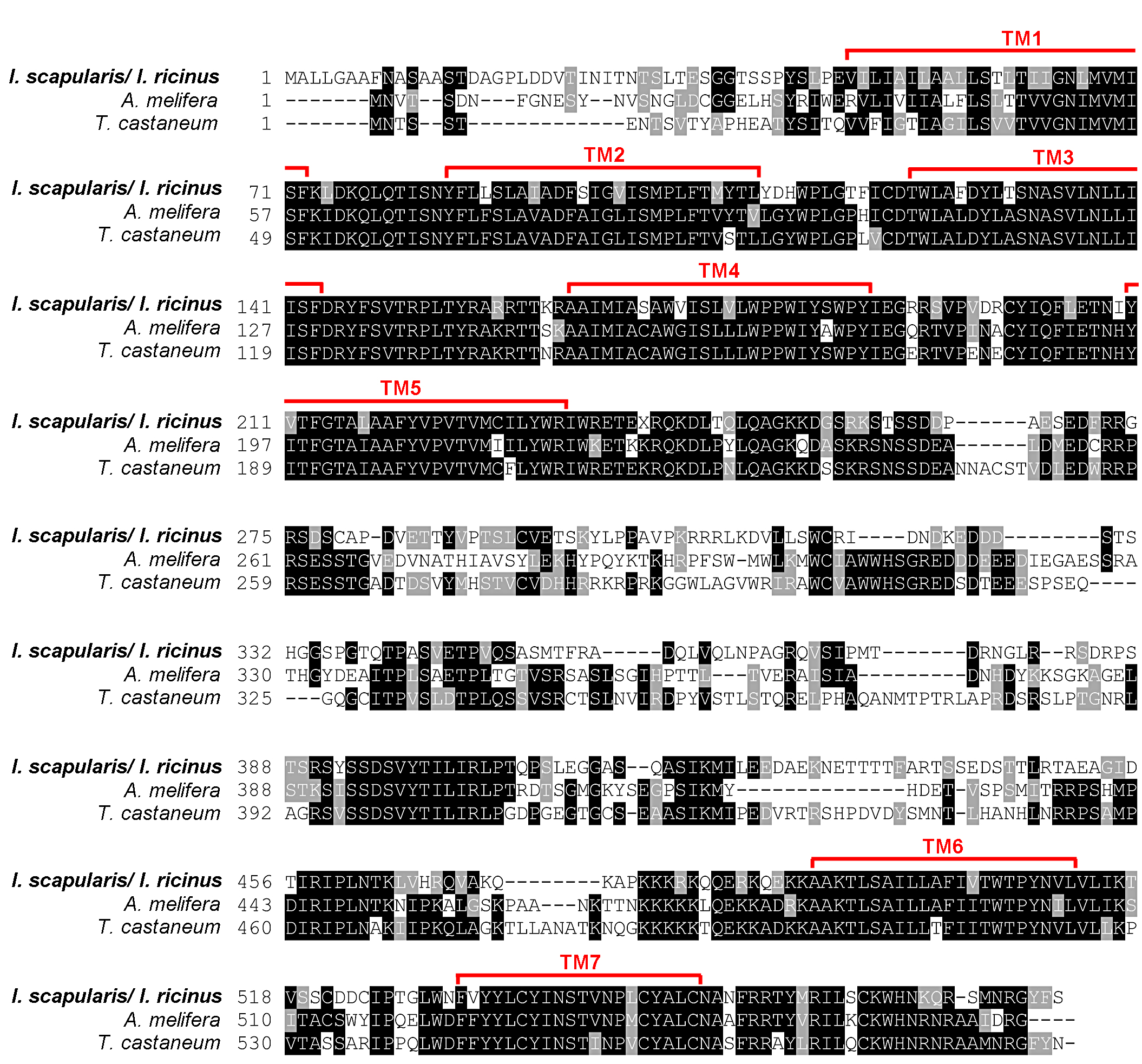
**

**Supplementary Figure S6.** Alignment of conceptual translations for mAChR-A-related sequences. The letters with gray background are similar amino acids, and letters with black background are identical amino acids in the sequence alignment using 50% majority rules. Seven conserved transmembrane segments of mAChR-A are indicated with red brackets above the alignment. The prediction of transmembrane segments was performed using TOPCONS software. For GenBank accession numbers see Fig. 5 caption in the main text.

**
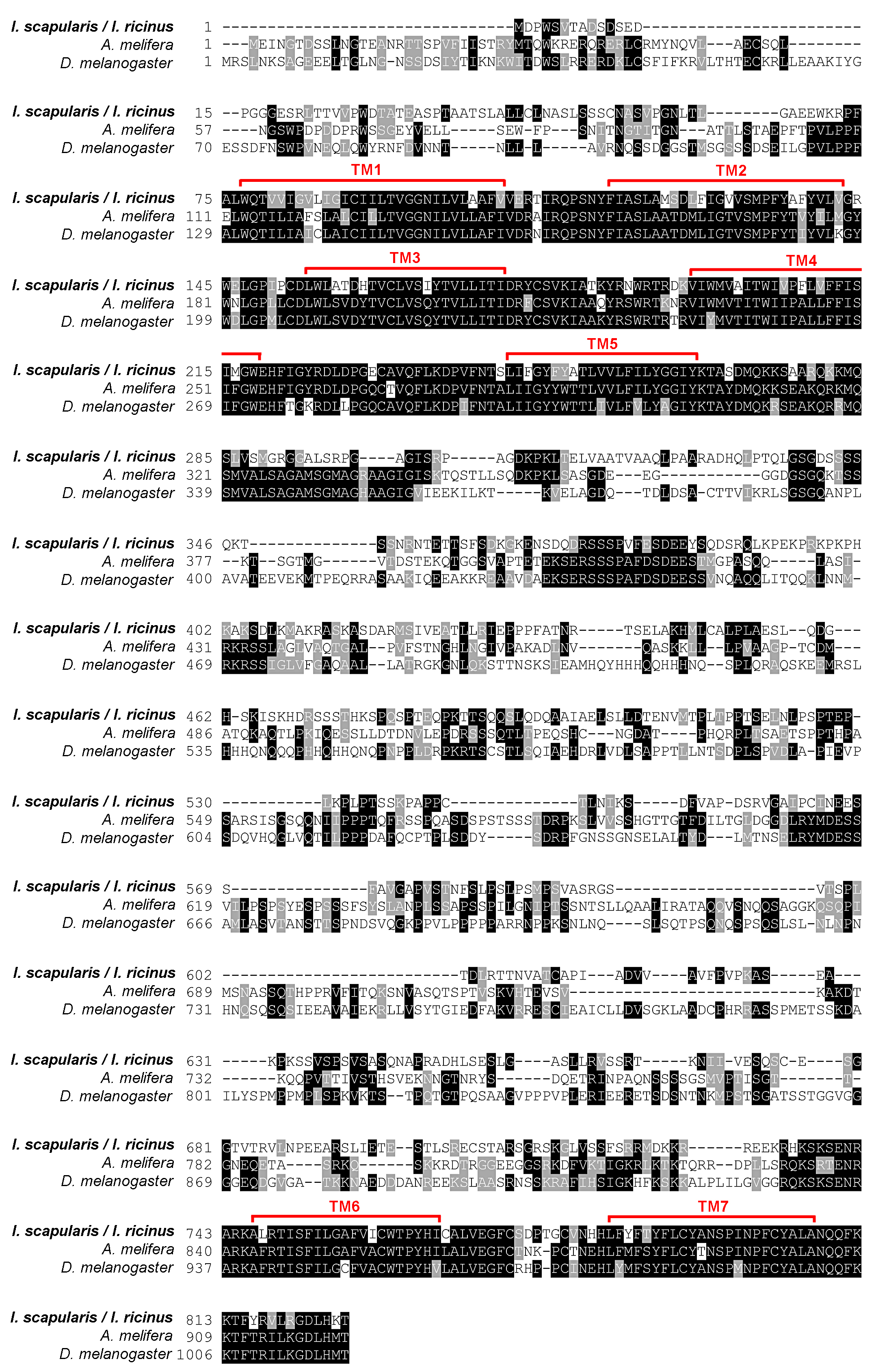
**

**Supplementary Figure S7.** Alignment of conceptual translations for mAChR-B related sequences. The letters with gray background are similar amino acids, and letters with black background are identical amino acids in the sequence alignment using 50% majority rules. Seven conserved transmembrane segments of mAChR-B are indicated with red brackets above the alignment. The prediction of transmembrane segments was performed using TOPCONS software. For GenBank accession numbers see Fig. 5 caption in the main text.


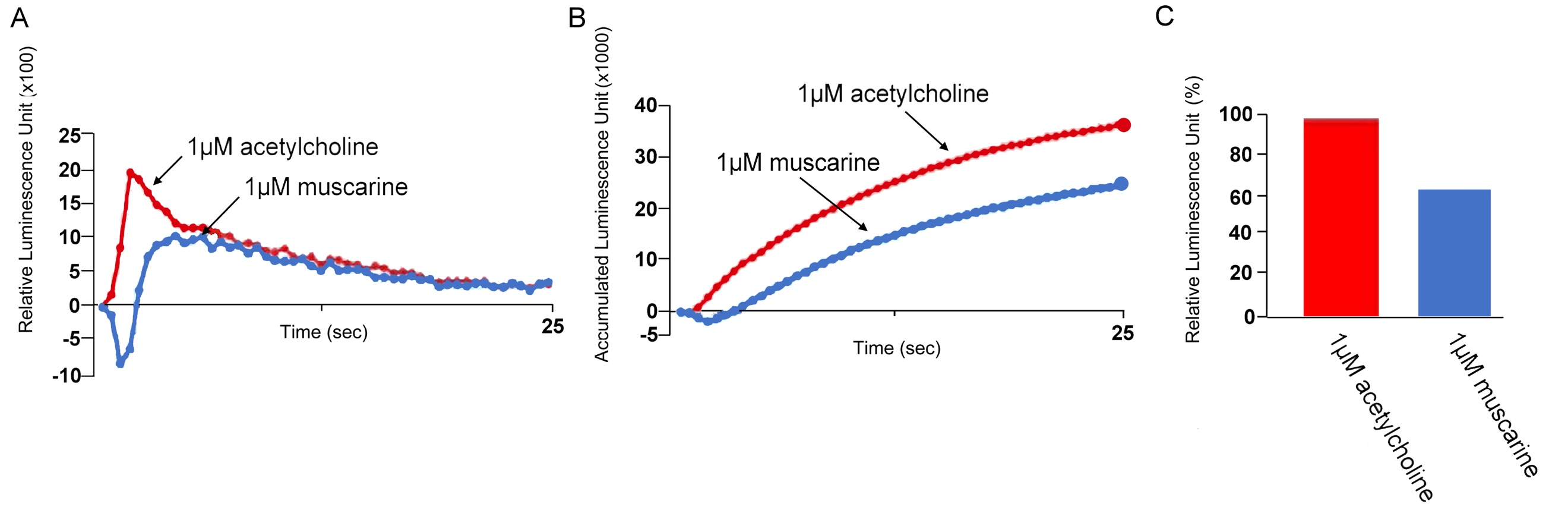


**Supplementary Figure S8.** Responses of mAChR-A-expressing CHO-K1 cells not expressing G_α15(16)_, to acetylcholine and muscarine. (A) 25 second responses to 1 μM acetylcholine and muscarine. (B) Integrated relative luminescent values of 25 second responses. (C) Relative luminescent responses of acetylcholine and muscarine. The highest value (acetylcholine) was set as 100.

**
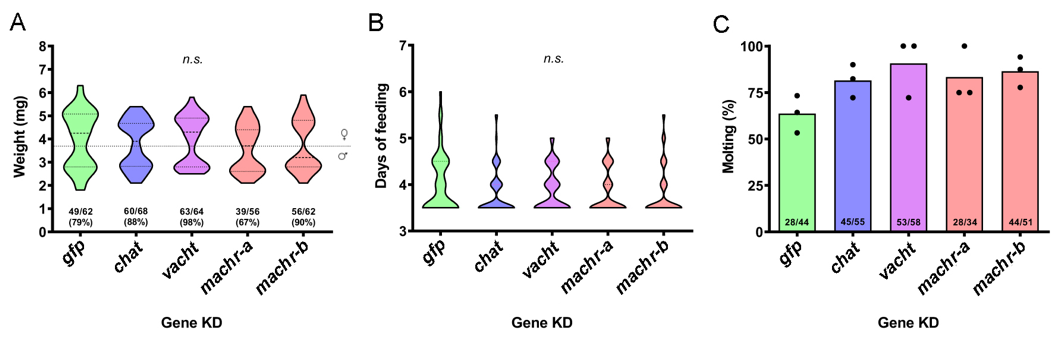
**

**Supplementary Figure S9. Silencing of genes in *I. ricinus* nymphs.** Silencing of *chat*, *vacht*, *machr-a*, and *machr-b* by RNA interference in nymphs did not interfere with nymph weight after feeding (A), the length of feeding (B), or molting from the engorged nymphs into adults (C). dsRNA for the green fluorescent protein (*gfp)* that does not exist in *Ixodes* genome sequence, was used as a negative control. (A) The numbers below the values indicate number and percent of engorged/placed nymphs. The dotted line shows the approximate border between females (above) and males (below) in the control group. (C) The numbers at the bottom of each column indicate number of molted nymphs. Each dot represents data from one mouse. KD- knock-down. The silenced ticks were fed on three individual mice (~20 injected nymphs per mouse). Statistics were performed using GraphPad Prism 4.0 (GraphPad Software, CA) employing One-way ANOVA Kruskal-Wallis tests and P < 0.05 were considered as significant (*n.s.* = not significant). For more detail on silencing techniques used, see Supplementary Methods online.

**
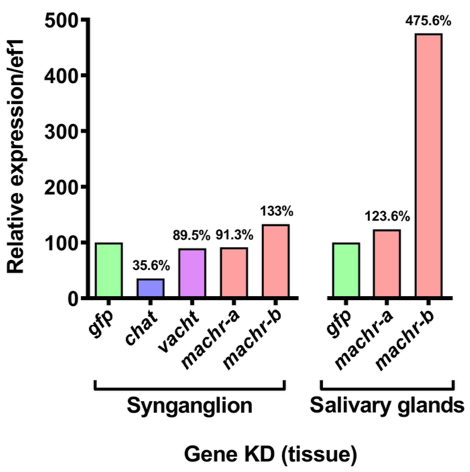
**

**Supplementary Figure S10.**  **Verification of gene silencing in *I. ricinus* nymphs.** Knock-down (KD) of *chat*, *vacht*, *machr-a*, and *machr-b* evoked by injection of dsRNA in nymphs. Notably decreased expression was only observed for *chat* in the synganglion. Each sample contained a pool of five engorged nymphs. RNA was reversibly transcribed using oligo-dT primers and qRT primers were designed outside the dsRNA region. The number above each column indicates the level of expression compared to dsRNA of the *gfp* control group (100%). Expression was normalized to the tick elongation factor gene (*ef*). For more information on silencing techniques used, see Supplementary Methods online.


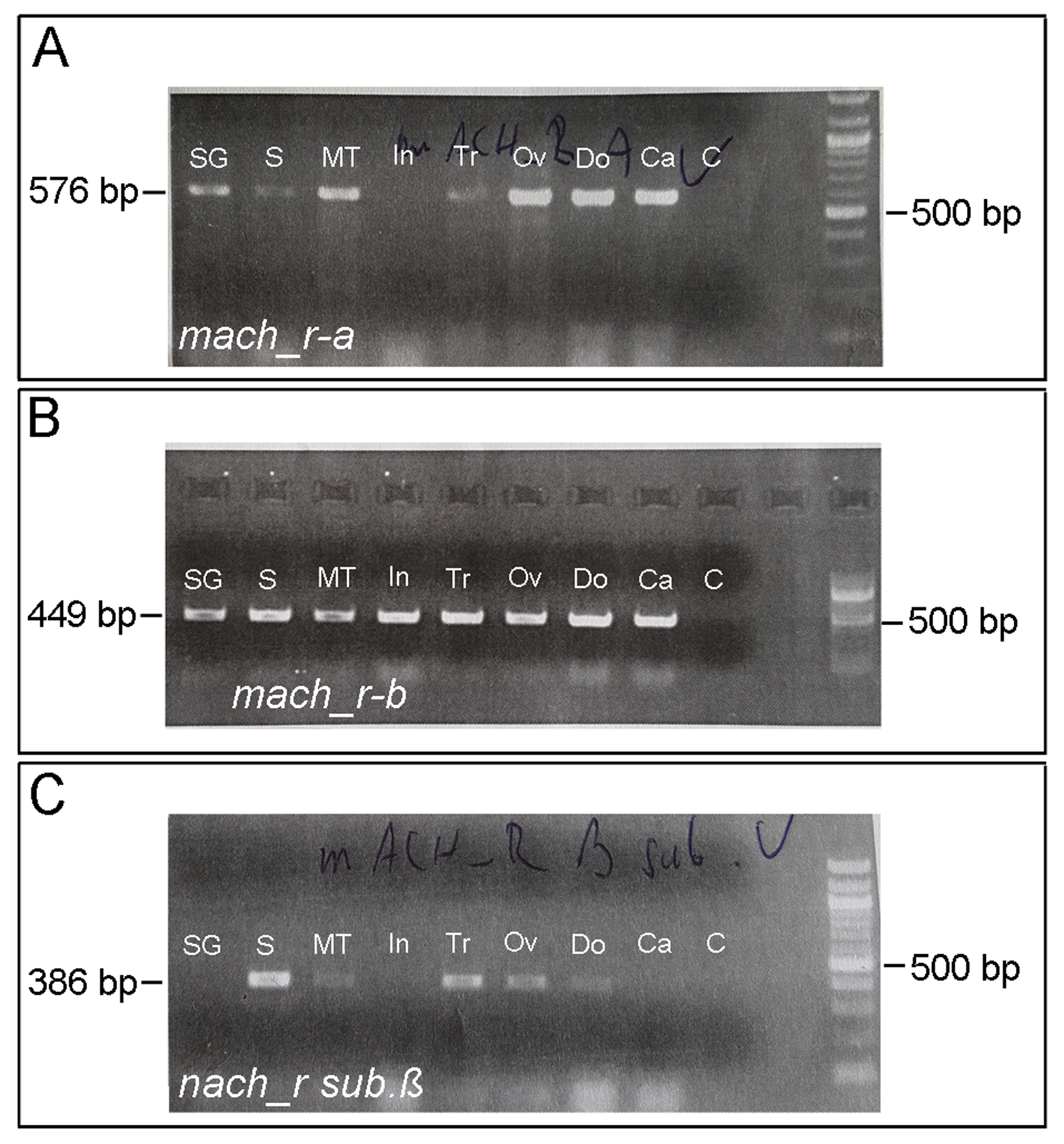


**Supplementary Figure S11.** Full-length gel images displayed in Figure 5 in the main text of tissue-specific PCR of mAChR-A (A), mAChR-B (B), and nAChR (subunit β) (C) in different tissues from partially-fed *I. ricinus* females. SG – salivary glands, S – synganglia, Mt – Malpighian tubules, In – intestine (midgut), Tr – tracheas, Ov – ovaries, Do – dorsum, Ca – carcass, C – control (without template).

**VIDEO LEGENDS**

**Video 1.** Z-stack confocal image of the posterior part of the *Ixodes* synganglion. The video highlights the opistosomal neurons (OsSG) and their putative projections entering the opistosomal nerves (See Fig. 2 E in main text). Note that the two following procedures, anti-*chat* *in situ* hybridization and anti-ChAT IHC were used for the specimen. The fluorescent (green) immunoreaction appears on the surface of the neuronal bodies while dark spots are visible in the deeper layers as a result of the *in situ* hybridization treatment. For the 3D rotation of the same image see Video 2 online.

**Video 2.** 3D reconstruction of the confocal image of the posterior part of the *Ixodes* synganglion. The video highlights the opistosomal neurons (OsSG) and their putative projections entering the opistosomal nerves (See Fig. 2 E in main text). Note that the two following procedures, anti-*chat* *in situ* hybridization and anti-ChAT IHC were used for the specimen. For the z-stack confocal image see Video 1 online.

**Video 3.** 3D reconstruction of the confocal image of the anterior part of the *Ixodes* salivary gland. The video highlights the ChAT-positive axons (cholinergic axons) reaching the type I acini salivary gland (See Fig. 3 B in main text). Note that cholinergic axons run along the main salivary duct and terminate exclusively within the individual type I acini.
