# Supplementary material for: Cholinergic axons regulate type I acini in salivary glands of Ixodes ricinus and Ixodes scapularis ticks
Source: Sci Rep. 2020 Sep 29;10:16054. doi: 10.1038/s41598-020-73077-1 (PMC7524744; doi:10.1038/s41598-020-73077-1)
Supplement: Supplementary file 2 — Supplementary Information 2. [file 41598_2020_73077_MOESM2_ESM.docx]

**Supplementary methods**

Cholinergic axons regulate type I acini in salivary glands of *Ixodes ricinus* and *Ixodes scapularis* ticks

Lourdes Mateos-Hernandéz^1†^, Baptiste Defaye^1,2†,§^, Marie Vancová^3,4^, Ondrej Hajdusek^3^, Radek Sima^3^, Yoonseong Park^6^, Houssam Attoui^7^ and Ladislav Šimo^1*^

^1^UMR BIPAR, INRAE, Ecole Nationale Vétérinaire d’Alfort, ANSES, Université Paris-Est, Maisons-Alfort, France

^2^Université de Limoges, Faculté de Pharmacie, Limoges, France

^3^Biology Centre, Institute of Parasitology, Czech Academy of Sciences, Ceske Budejovice, Czech Republic

^4^Faculty of Science, University of South Bohemia, Ceske Budejovice, Czech Republic

^6^Department of Entomology, Kansas State University, 123 Waters Hall, Manhattan, KS, USA

^7^UMR Virologie, INRAE, Ecole Nationale Vétérinaire d’Alfort, ANSES, Université Paris-Est, Maisons-Alfort,

France

† Equal contribution

§ Current address: UMR SPE 6134 CNRS, Université de Corte Pascal Paoli, Corse

* Corresponding author: [ladislav.simo@vet-alfort.fr](mailto:ladislav.simo@vet-alfort.fr)

**mAChR-B functional assays**

Full-length ORFs of mAChR-B were inserted into the expression plasmid pcDNA3/Zeo(+) (Invitrogen). To monitor intracellular calcium mobilization-triggered bioluminescence upon receptor activation, we used CHO-K1 cells, or CHO-K1 cells expressing the G_α15(16)_ subunit as described in the Material and methods in the main text.

To assess cAMP inhibition upon mAChR-B activation, we used the non-lytic GloSensor cyclic AMP assay system (Promega)^1^. HEK293 cells (Sigma) were co-transfected with the pcDNA3/Zeo(+)/mAChR-B and the GloSensor-expressing plasmid (Promega). Cells were pre-equilibrated with GloSensor reagent (Promega) for 2 h at RT. We also incubated cells (2 h) with or without 75 μM chloroquine (Sigma) to block the IP_3_ receptor and subsequent putative endogenous receptors linked to Ca^++^ pathways, that could potentially interfere with our assay.

In the 96-well microplate the cells (~10^5^ in 50 μL in each well) were pre-treated, 5 min at RT, with different doses of ACh (in 50 μl). Subsequently 50 μL of forskolin (10 μM final concentration in each well) was injected and the luminescence was monitored at ~ 1 min intervals for 20 min. Relative luminescence at 15 min after exposure to different doses of the ACh ligand was normalized to the highest response (cells treated with 10 μM forskolin only) in each plate after subtracting background. Note that no calcium mobilization or cAMP inhibition responses were observed for *Ixodes* mAChR-B in our reporter systems.

**RNA interference in *I. ricinus* nymphs**

*Ixodes ricinus* nymphs were obtained from the Biology Centre, Institute of Parasitology breeding facility, at the Czech Academy of Sciences, Czech Republic, and maintained in glass vials in humid chambers at 24°C. All laboratory animals were treated in accordance with the Animal Protection Law of the Czech Republic No. 246/1992 Sb., ethics approval No. 102/2016.

Using PCR, 496-544 bp fragments of *I. ricinus* *chat*, *vacht*, *machr-a*, and *machr-b* were amplified from *I. ricinus* cDNA and cloned into the pll10 vector with two T7 promoters in reverse orientations^2^, using primers (listed in Table 1 below) containing additional restriction sites *ApaI* and *XbaI*. The dsRNA was synthesized as described previously^3^. dsRNA (32.2 nl of 3 μg/μl) was injected into the hemocoel nymphs through the coxa of the third pair of legs using a Nanoinject II (Drummond). After three days of rest in a humid chamber at room temperature, ticks were fed on 6-week-old BALB-C mice (Velaz, Czech Republic). Gene silencing efficacy was verified by independent feeding experiments in a pool of five synganglia or salivary glands dissected from fully-engorged nymphs by qRT-PCR according to^4^. Tick elongation factor was used as a house-keeping gene.

To assess the effect of gene silencing on tick feeding and reproduction, 60 nymphs were injected with the dsRNA and fed on three individual mice (20 nymphs per mouse). Weight after feeding, length (days) of feeding, and molting of nymphs into adults were observed. Statistical significance of differences was analyzed using GraphPad Prism 4.0 (GraphPad Software, CA) employing One-way ANOVA Kruskal-Wallis tests, where P < 0.05 was considered as significant.

| **Method** | **Target** | **Sequence (3´🡪 5´)** | **Amplicon (bp)** |
| --- | --- | --- | --- |
| RNAi | *chat* | ATGGGCCCGGAATTCCTCGACTTCGTGC | 542 |
|  |  | ATTCTAGACGTGTAGCCTGGCCATCTCG |  |
|  | *vacht* | ATGGGCCCCTCCAAAGCCATCGTCCAGC | 496 |
|  |  | ATTCTAGAGATGAAGAGGCGCCAGATGG |  |
|  | *machr-a* | ATGGGCCCACGACAGCACCAGCCATGG | 544 |
|  |  | ATTCTAGACGTCCAGGTGACGATGAAGG |  |
|  | *machr-b* | ATGGGCCCCCATGCCTTCCGTAGCTAGC | 527 |
|  |  | ATTCTAGACGTCCAGCAGATAACGAACG |  |
| qRT-PCR | *chat* | CAAGTCTTACGCCGACTTCC | 126 |
|  |  | GGGCAGATGTTGTAGGAACAG |  |
|  | *vacht* | AACAGAGGCCTCCCCATAA | 103 |
|  |  | TCCTCTTCGGAACTGGTGAT |  |
|  | *machr-a* | GTCTCCTCGTGTGACGACTG | 131 |
|  |  | CGCATGTATGTCCTCCTGAA |  |
|  | *machr-b* | GGGTGCGTTAATCACCATCT | 131 |
|  |  | TCACCTCGCAGTACCCTGTA |  |
|  | Tick *elongation factor* | acgaggctctgacggaag | 81 |
|  |  | cacgacgcaactccttcac |  |

**Supplementary Table 2.** List of primers used in RNAi experiments. Restriction sites for *ApaI*/*XbaI* are underlined.

**REFERENCES:**

1. Šimo, L., Koči, J., Žitňan, D. & Park, Y. Evidence for D1 Dopamine Receptor Activation by a Paracrine Signal of Dopamine in Tick Salivary Glands. PLoS One 6, (2011).

2. Levashina, E. A. et al. Conserved Role of a Complement-like Protein in Phagocytosis Revealed by dsRNA Knockout in Cultured Cells of the Mosquito, Anopheles gambiae. Cell 104, 709–718 (2001).

3. Hajdusek, O. et al. Knockdown of proteins involved in iron metabolism limits tick reproduction and development. Proceedings of the National Academy of Sciences of the United States of America 106, (2009).

4. Honig Mondekova, H. et al. Characterization of Ixodes ricinus Fibrinogen-Related Proteins (Ixoderins) Discloses Their Function in the Tick Innate Immunity. Frontiers in Cellular and Infection Microbiology (2017) doi:10.3389/fcimb.2017.00509.
